# RAS-protein activation but not mutation status is an outcome predictor and unifying therapeutic target for high-risk acute lymphoblastic leukemia

**DOI:** 10.1038/s41388-020-01567-7

**Published:** 2020-11-27

**Authors:** David Koschut, Debleena Ray, Zhenhua Li, Emanuela Giarin, Jürgen Groet, Ivan Alić, Shirley Kow-Yin Kham, Wee Joo Chng, Hany Ariffin, David M. Weinstock, Allen Eng-Juh Yeoh, Giuseppe Basso, Dean Nižetić

**Affiliations:** 1 Lee Kong Chian School of Medicine, Nanyang Technological University, Singapore, Singapore; 2 Department of Paediatrics, Yong Loo Lin School of Medicine, National University of Singapore, Singapore, Singapore; 3 Department of Women’s and Children’s Health (SDB), Hematology-Oncology Laboratory, University of Padua, Padua, Italy; 4 The Blizard Institute, Barts and The London School of Medicine and Dentistry, Queen Mary University of London, London, United Kingdom; 5 Department of Anatomy, Histology and Embryology, Faculty of Veterinary Medicine, University of Zagreb, Zagreb, Croatia; 6 National University Cancer Institute, Singapore, Singapore; 7 University of Malaya Medical Centre, University of Malaya, Kuala Lumpur, Malaysia; 8 Department of Medical Oncology, Dana-Farber Cancer Institute, Harvard Medical School, Boston, MA USA; 9 Italian Institute for Genomic Medicine, Turin, Italy

**Keywords:** Acute lymphocytic leukaemia, Molecular biology

## Abstract

Leukemias are routinely sub-typed for risk/outcome prediction and therapy choice using acquired mutations and chromosomal rearrangements. Down syndrome acute lymphoblastic leukemia (DS‐ALL) is characterized by high frequency of *CRLF2*‐rearrangements, *JAK2*‐mutations, or RAS‐pathway mutations. Intriguingly, *JAK2* and *RAS*-mutations are mutually exclusive in leukemic sub‐clones, causing dichotomy in therapeutic target choices. We prove in a cell model that elevated CRLF2 in combination with constitutionally active JAK2 is sufficient to activate wtRAS. On primary clinical DS‐ALL samples, we show that wtRAS-activation is an obligatory consequence of mutated/hyperphosphorylated JAK2. We further prove that CRLF2-ligand TSLP boosts the direct binding of active PTPN11 to wtRAS, providing the molecular mechanism for the wtRAS activation. Pre‐inhibition of RAS or PTPN11, but not of PI3K or JAK‐signaling, prevented TSLP‐induced RAS‐GTP boost. Cytotoxicity assays on primary clinical DS‐ALL samples demonstrated that, regardless of mutation status, high-risk leukemic cells could only be killed using RAS‐inhibitor or PTPN11-inhibitor, but not PI3K/JAK‐inhibitors, suggesting a unified treatment target for up to 80% of DS‐ALL. Importantly, protein activities-based principal-component-analysis multivariate clusters analyzed for independent outcome prediction using Cox proportional-hazards model showed that protein‐activity (but not mutation-status) was independently predictive of outcome, demanding a paradigm-shift in patient‐stratification strategy for precision therapy in high-risk ALL.

## Introduction

Acute lymphoblastic leukemia (ALL) is the most common malignancy and cancer-related cause of death at pediatric age [1, 2]. Despite a considerable success rate of standard chemotherapy treatments, as many as 10–15% of children with ALL have recurrent disease (relapses) [3, 4]. Patients with high-risk (HR) forms of ALL show increased incidence of relapses, poorer prognosis and lower overall 5-year survival rates following relapse [5]. Recently, significant progress has been achieved in understanding the mechanistic consequences of individual pathways affected in HR-ALL, and the resulting selection of therapeutic targets leading to clinical trials using pathway-specific drugs, such as JAK/STAT inhibitors [6]. Recent detailed studies of the evolution of acquired genomic changes in ALL identified certain sub-types as being particularly HR forms [7, 8]. Among these are hypodiploid ALL [9], Philadelphia chromosome-like (Ph-like) type (defined as a type of ALL with the genomic profile similar to that of the Ph+ ALL) [8, 10, 11], ALL with an intrachromosomal amplification of chromosome 21 (iAMP21) [12, 13], and ALL in children with Down syndrome (DS-ALL) [14, 15].

The acquired mutations landscape does not find a unifying profile that distinguishes HR childhood ALL from non-HR childhood ALL, suggesting the need for individualized therapy approach [16] preceded by individual patient sub-type assignment based on the mutational profile analysis. While Ph-like ALL has a high incidence (60%) of genomic rearrangements leading to an increased expression of the receptor to the cytokine TSLP, CRLF2 [17], and more than half of these have mutations in JAK and IL7R pathway - including constitutionally activating JAK2 mutations [11, 18, 19], less than 10% of Ph-like ALL also acquire RAS/MAPK pathway mutations. DS-ALL is distinguished by the similarly high presence of both *CRLF2*-rearrangements (60%) (with *JAK2* mutations at 32%), with a higher proportion of RAS-MAPK pathway mutations (36%) [20, 21]. Intriguingly, a near complete mutual exclusion between *JAK2* and *RAS* mutations in diagnosis samples, or individual sub-clones of relapse samples of DS-ALL is repeatedly observable [20, 21].

We hypothesized that the reason for this mutual exclusion is that increased CRLF2-levels in combination with JAK2 activation could be sufficient to activate wild-type (wt) RAS protein in the absence of *RAS* mutations.

## Materials and methods

### Cell culture and cell viability

Ba/F3 (Cat.#RCB0805), a murine IL-3 dependent pro-B cell line, was obtained from RIKEN BioResource Center (Tsukuba, JP) and MUTZ-5 (Cat.#ACC490), a human B cell precursor leukemia cell line established at relapse, was obtained from the Leibniz Institute DSMZ (Braunschweig, DE); authenticated via multiplex PCR of minisatellite markers. Mycoplasma-free cells were routinely passaged (passage range for shown experiments: 15–35) according to the respective cell bank recommendations. Handling of primary patient samples is described in detail in Supplementary Material.

Cell count and cell viability (percentage of acridine orange-positive cells not stained by 4′,6-diamidino-2-phenylindole (DAPI) were determined in an NC-250 automated cell-analyzer (ChemoMetec, Allerod, DK).

### Patient samples

Surplus clinical or archived clinical material for peripheral blood/bone marrow samples of DS-ALL and non-DS ALL patients was collected by the tissue bank of the Italian Association for Paediatric Haematology-Oncology (AIEOP). In accordance with the Declaration of Helsinki, informed written consent was obtained by the tissue bank for all subjects. Samples were processed and stored in the tissue bank at The Blizard Institute, which is licensed for tissue storage and monitored by UK-Human Tissue Authority. Detailed clinical description of studied DS-ALLs and Non-DS B-ALLs is available in Supplementary Table S1. Detailed cytogenetics was available in 12 cases.

MS2003/2010 cohort [22, 23] RNA-seq data was submitted to the European Genome-phenome Archive (Accession# EGAS00001001858).

### RAS activity assays

Cells were left uninduced or induced with human TSLP at 37 °C. Whenever indicated, DMSO or inhibitors were added for 3 h before TSLP-induction. Cells were lysed on ice at 1000 cells/µL lysis buffer according to the manufacturer’s protocol of the active RAS detection kit (Cat.#8821; Cell Signaling Technology, Danvers, US). Total protein concentrations of samples were measured using a BCA protein-assay kit (Cat.#23225; ThermoFisher Scientific, Waltham, US). 50 µg total protein was loaded per column of the active RAS detection kit for Western blot (WB). In the RAS activation assay kit for ELISA (Cat.#17-497; EMD Millipore, Burlington, US), 12 µg total protein was used at 100 ng/µL and the RAS-GTP pull-down was measured using a Synergy H1 plate reader (BioTek, Winooski, US) in luminescent mode.

For methods on proximity ligation assay (PLA), principal-component-analysis (PCA), statistical analysis, as well as lists of antibodies/chemicals, and standard protocols for the sequencing, SDS-PAGE/WB, phospho-protein antibody-microarray, and transduction please see “Supplementary Methods”.

## Results

### CRLF2 and JAK2mut co-expression is sufficient to activate RAS in Ba/F3 cells

**Fig. 1:**
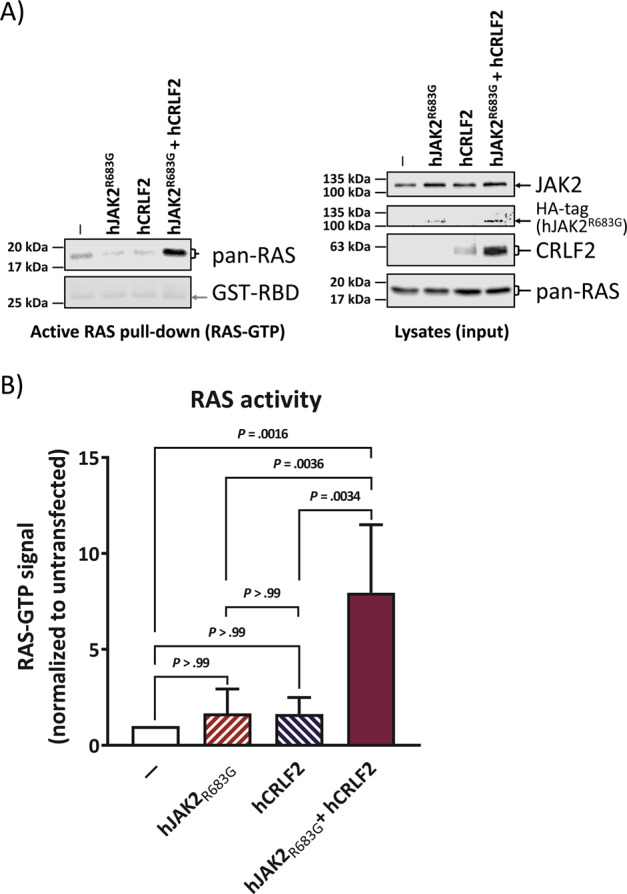
Combination of CRLF2 overexpression and constitutively active JAK2 is sufficient for wt RAS activation. WB analysis of the murine pro B cell line Ba/F3. Cells were stably transfected with human JAK2_R683G_ and/or human CRLF2 (see Supplementary Figs. S1C and S1D) and cultured in IL-3-containing medium. All cells were then starved from IL-3 and cells were lysed. Each cell lysate was split up for analysis in RAS-GTP pull-down assay and for total proteins. An SDS-PAGE followed by WB was performed. **a** Left-hand side blot shows the RAS-GTP (activated RAS) pull-down while the right-hand side blots show whole cell lysates of the same samples. Antibody-targets are labeled on the right side of each image with black arrows marking the respective protein band; the antibody against HA-tag shows the expression of the human JAK2 construct. The experiment was repeated four times independently. **b** Quantification of **a** for active RAS (RAS-GTP) normalized to its level in untransfected cells. Error bars are SD and *P* values were determined in one-way ANOVA and post-hoc Bonferroni multiple comparison.

We hypothesized that increased CRLF2-level in combination with a mutation in JAK2 pathway genes could be sufficient to activate wtRAS protein in the absence of *RAS* mutations, as a mechanism to explain the mutual exclusion of *JAK2* and RAS/MAPK mutations in DS-ALL. The level of RAS activity is generally assessed using a pull-down assay whereby the (activated) RAS-GTP is captured by virtue of its high affinity to RAS-binding-domain (RBD) of RAF proteins. In order to observe the effects of elevated CRLF2 signaling on the activation of RAS, we stably integrated a human *CRLF2* overexpression construct [24] into the mouse pre-B-cell line Ba/F3. This alteration did not increase the level of pulled-down RAS-GTP (Fig. 1a) and neither did the stable overexpression of hJAK2_R683G_ [24], the most prevalent specific activating JAK2 mutation in DS-ALL and Ph-like-ALL. However, when both of these alterations were combined, eight fold higher RAS-GTP level was measured, in the absence of cytokines (Fig. 1b, post-hoc Bonferroni *p* values are listed in Supplementary Table S2). An independent set of Ba/F3 lines, in which *CRLF2* was transduced first, confirms that this increased RAS-activity is not due to variations in CRLF2 overexpression levels within the lines (Supplementary Fig. S1F). Growth of Ba/F3 cells depends on IL-3 (Supplementary Fig. S1A), which induces wtJAK2 phosphorylation [25], and interestingly we found that it also activates RAS (Supplementary Fig. S1B). The cells with combined CRLF2 and JAK2_R683G_ overexpression were the only ones in this series that grew in a cytokine-independent manner (Supplementary Fig. S1E), as also previously observed [24]. This proves that increased CRLF2-expression together with activated JAK2 is sufficient to activate wtRAS, and this coincides with the transition to cytokine-independent growth.

### TSLP-inducible RAS activity in absence of RAS mutations is a feature of human CRLF2 rearranged B-ALL

**Fig. 2:**
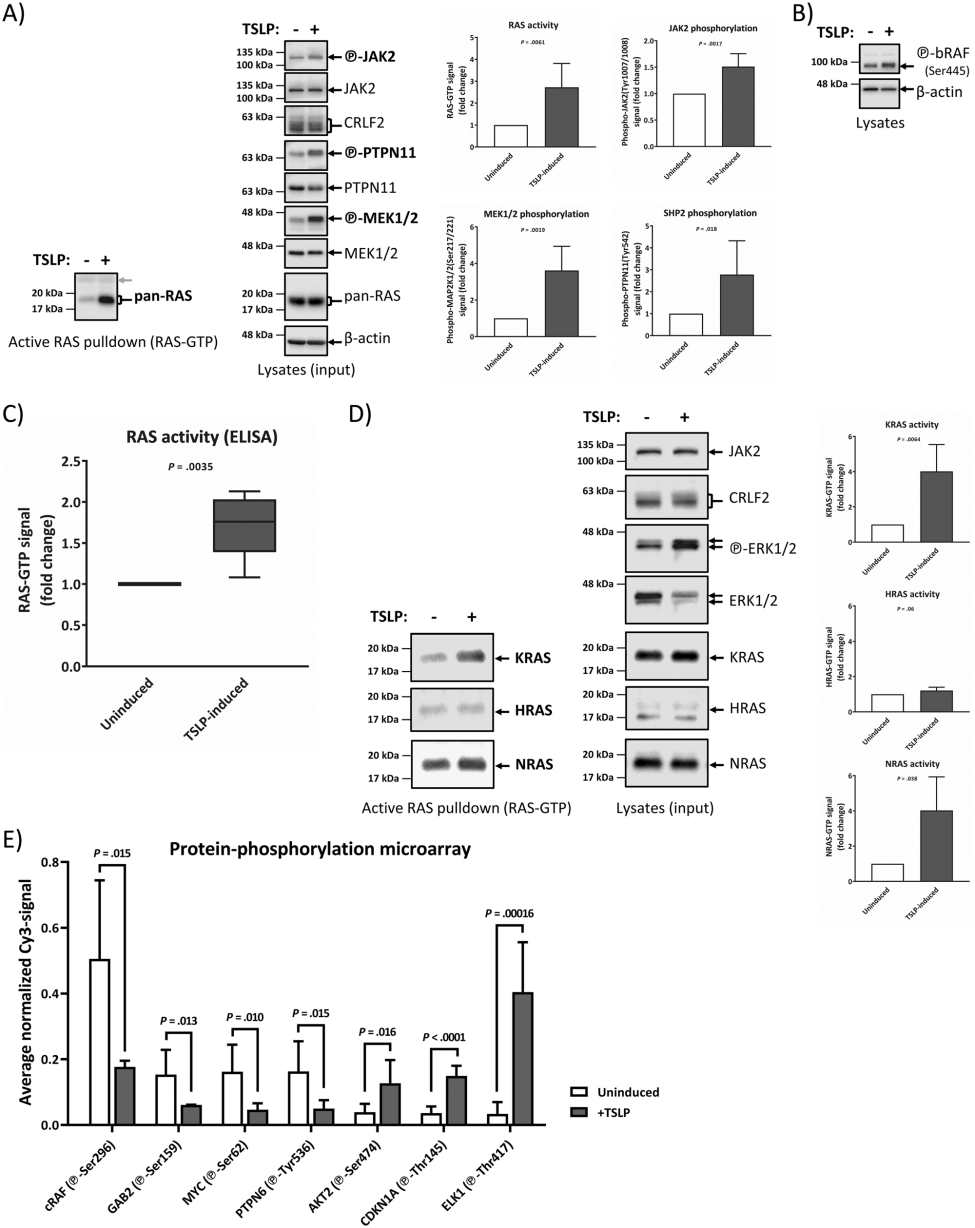
Human Ph-like B-ALL (spontaneous CRLF2-rearrangment and JAK2_R683G_-mutation) cells activate wtRAS and RAS-interacting proteins upon TSLP-induction. MUTZ-5 cells were stimulated with 20 ng/mL hTSLP (maximal effective TSLP-concentration, Supplementary Fig. S4B) for 10 min before lysis. Each lysate was split up for analysis in RAS-GTP pull-down assay and for WB. **a** An SDS-PAGE followed by WB was performed. To assess the total protein and phosphorylated protein amounts on the same PVDF-membrane, each membrane part was stripped and reprobed with new antibodies. RAS-GTP pull-down blots are on the left side while the right-hand side blots show whole cell lysates of the same samples. The gray arrow shows the unspecific signal of the GST-RAS binding domain (RBD) used in the active RAS pull-down assay acting as a loading control. The experiment was repeated five times independently and the graphs show the quantification for active RAS (RAS-GTP), phospho-MEK1/2, phospho-JAK2, and phospho-PTPN11. Beta-actin and total protein signals were used as a loading control to normalize samples. **b** A blot separate from **a** demonstrates the TSLP-inducibility of RAS-effector bRAF. **c** Quantification of five independent ELISA experiments in which RAS-GTP in MUTZ-5 cells was measured using a different, ELISA-specific active-RAS pull-down assay. **d** MUTZ-5 cells were probed for the activation of KRAS-GTP, HRAS-GTP, or NRAS-GTP isoforms (left side). The blots on the right show the total expression of the respective RAS proteins and the graphs show the average signal fold-change for KRAS-GTP, HRAS-GTP and NRAS-GTP (*N* = 4, means ± SD). *P* values were calculated using Student’s *T* test and adjusted with a Bonferroni-correction for sequential multiple-comparison. **e** Whole, non-denatured lysate from uninduced or TSLP-induced MUTZ-5 cells was subjected to an antibody-microarray. The graph shows relevant, most statistically significant changes in protein-phosphorylations, a heatmap-overview for all analyzed protein-phosphorylations can be found in Supplementary Fig. S4C. (*N* = 6, means ± SD). *P* values were calculated using Student’s *T* test (Bonferroni-correction for sequential multiple comparison can be found in Supplementary Table S2).

In order to prove the observations from Fig. 1 in human ALL cells, we selected a B-ALL cell line that harbors identical changes as our double-transfected model line in Fig. 1. The B cell precursor leukemia cell line MUTZ-5 from a relapsed Philadelphia-like B-ALL patient features a *CRLF2*-translocation leading to wt CRLF2 overexpression, as well as the *JAK2*_R683G_ mutation, and the absence of mutations in any RAS-MAPK pathway genes [26]. The absence of *RAS* mutations in the MUTZ-5 cells grown in our cultures was confirmed by performing standard Sanger DNA-sequencing of PCR-amplicons from genomic DNA, encompassing all exons of *KRAS*, *NRAS* and *HRAS* genes (Supplementary Table S3). We detected the presence of activated RAS in these cells by RAF-RBD pull-down of RAS-GTP (Fig. 2a), which was tripled upon a 10 min induction with the CRLF2-ligand TSLP. Similar results were reproduced using an ELISA-based RAS-pull-down (Fig. 2c). Both immediate upstream (PTPN11) and immediate downstream (MEK1/2, bRAF) components of the RAS/MAPK pathway were also induced by TSLP induction (Fig. 2a, b). The direct binding of activated RAS and bRAF proteins expressed in these cells (Supplementary Fig. S2A) was further validated via PLA (Supplementary Fig. S2B), as was RAS and phospho-PTPN11 interaction in the Ba/F3 model (Supplementary Fig. S3B). Both KRAS and NRAS, but not HRAS, isoforms showed increased activity after TSLP-induction (Fig. 2c). Interestingly, the genes for the same two isoforms (*KRAS* and *NRAS*), but not *HRAS*, acquire mutations in B-ALL [12, 20]. Therefore, we conclude that the RAS isoform activity pattern of TSLP-inducibility in wtRAS leukemia cells matches the isoforms that acquire mutations in RAS-mutated leukemia cases. Furthermore, we traced TSLP-signaling throughout cellular pathways in 68 individual protein-phosphorylation sites via an antibody-based phospho-array (Supplementary Fig. S4C). The phospho-array confirmed the increased phosphorylation observed for denatured proteins in WB for STAT5A, ERK1/2, MEK1, and JAK2 on their respective native epitopes (Supplementary Fig. S4C) while the most statistically significant results demonstrate additional TSLP-effects by increasing activating phosphorylations (AKT2, CDKN1A, ELK1) but also by downregulating pathway-inhibiting phosphorylations (cRAF(Ser296), GAB2, MYC, PTPN6) (Fig. 2e).

### RAS inhibitor can significantly block the growth of human B-ALL Ph-like wtRAS cells

**Fig. 3:**
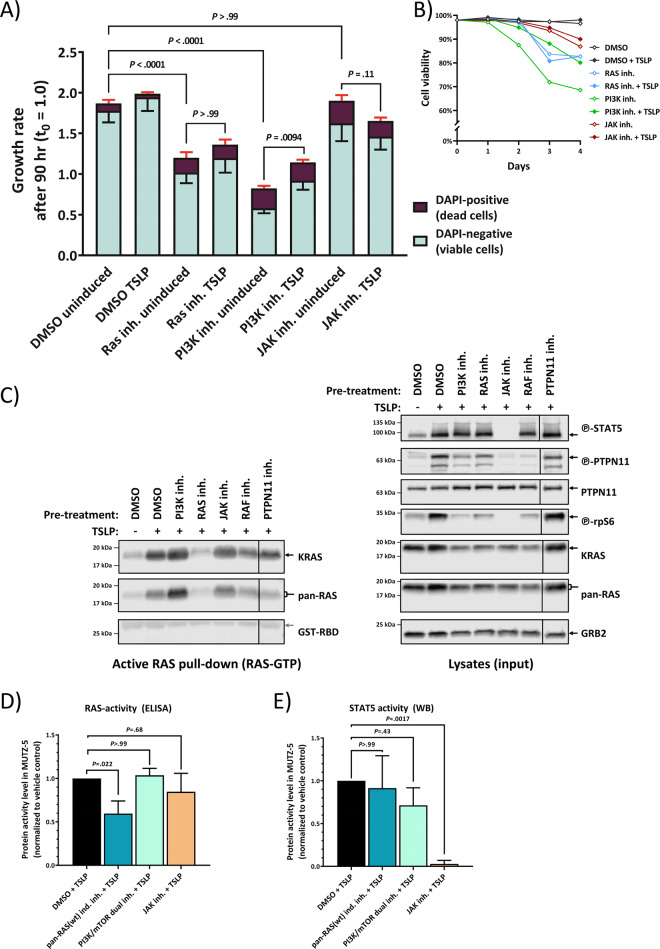
Inhibition of RAS stops wt-RAS Philadelphia-like ALL cell growth in the presence of TSLP. **a** MUTZ-5 cells were seeded at 6.5 × 10^5^/mL density and cultured over 4 days with either 0.5% DMSO (vehicle control), 50 µM Salirasib (indirect Pan-RAS inh.), 10 µM PI-103 (PI3K/mTOR dual inh.), or 5 µM Ruxolitinib (JAK inh.), each in absence or presence of 20 ng/mL human TSLP. Cell count and viability was determined in an NC-250 automated cell counter daily. The stacked-bar graph on the left side shows the growth rate after the 90 h timepoint, averaged from two independent experiments, each with triplicate wells. Red error bars are SD from the dead cell fraction while the black error bars show the SD of the viable cells. *P* values were calculated in one-way ANOVA from the total cell growth rate and adjusted in a post-hoc Bonferroni multiple comparison. Only relevant *P* values are shown in the graph, for a complete list see Supplementary Table S2. **b** The graph shows the cell viability of the experiment in **a** over time. **c** MUTZ-5 cells were pre-treated for 2 h with either 0.5% DMSO (vehicle control), 10 µM PI-103 (PI3K/mTOR dual inh.), 50 µM Salirasib (indirect pan-RAS inh.), 5 µM Ruxolitinib (JAK inh.), 50 µM Vemurafenib (Pan-Raf inh.), or 25 µM II-B08 (PTPN11 inh.), and then stimulated with 20 ng/mL human TSLP for 10 min followed by cell lysis. Each lysate sample was split up for analysis in RAS-GTP pull-down assay and for total protein signal. RAS-GTP pull-down (left) and lysate samples (right) were loaded on separate gels. An SDS-PAGE followed by WB was performed. To assess the total protein and phosphorylated protein amounts on the same PVDF-membrane, membranes were stripped and reprobed with new antibodies. Antibody-targets are labeled on the right side of each image with black arrows indicating the respective protein band. **d** MUTZ-5 cells were treated with 50 µM Salirasib (pan-RAS-inhibitor), 10 µM PI-103 (PI3K/mTOR dual inhibitor), or 5 µM Ruxolitinib (JAK-inhibitor) like in **c** after which the RAS-GTP levels were measured in ELISA. *N* = 3 independent experiments, bar graph shows means ± SD. **e** MUTZ-5 cells were treated as in **d** and STAT5 activity was determined via Western blot. *N* = 3 independent experiments, bar graph shows means ± SD. *P* values for **d** and **e** were calculated in one-way ANOVA and post-hoc Bonferroni multiple comparison.

We next examined to what extent the direct RAS activation in wtRAS leukemic cells affects the cell growth and viability. We tracked the cell count and cell viability of MUTZ-5 cells after treatment with pan-wtRAS-inhibitor and in comparison to treatments with other compounds that have been previously reported to induce dose-dependent cytotoxicity in MUTZ-5 [27], some of which are currently in clinical trials for Ph-like ALL [6]. After 4 days treatment with pan-wtRAS inhibitor the growth and viability of MUTZ-5 cells were significantly reduced (Fig. 3a, b), and this was not affected by the presence of TSLP. In comparison, the dual PI3K/mTOR inhibitor also significantly reduced the growth and viability of MUTZ-5 cells, but this inhibitory effect could be partially counteracted by the TSLP-induction (Fig. 3a). Both of these compounds, at the concentrations used (tested to achieve high efficacy on the respective main pathway target in WB, Fig. 3c), showed a much stronger inhibitory effect on cell growth than the JAK inhibitor (Fig. 3a), despite the observation that this concentration of JAK-inhibitor, which blocks almost all STAT5-signaling (Fig. 3c, e), showed the strongest inhibition of TSLP-induced phosphorylation of MEK1/2, PTPN11, ERK1/2 and rpS6 (Fig. 3c, right-hand side blots). However, neither the JAK inhibitor, nor the PI3K/mTOR inhibitor could block the wtRAS activation by TSLP as shown in WB (Fig. 3c, left-hand side blots) and ELISA (Fig. 3d). For the JAK2 inhibitor this might be explained by its failure to reduce the direct interaction between RAS and PTPN11 in PLA (Supplementary Fig. S2E). In contrast, the pan-wtRAS-inhibitor significantly blocked the TSLP-induced RAS-activity (Fig. 3c, left-hand side blot) and ELISA (Fig. 3d). Moreover, the pre-inhibition of RAS-direct interacting proteins (RAF and PTPN11) also reduced TSLP-induced wtRAS boost in human Ph-ALL cells (Fig. 3c). Combined, our data suggest that TSLP-activation of RAS in the absence of RAS mutations drives B-ALL cell growth, and represents an independent drug target, in addition to the PI3K/mTOR and JAK/STAT pathway targets.

### DS-ALL patients differ in the level of activity and inducibility of RAS, independently of RAS mutations

**Fig. 4:**
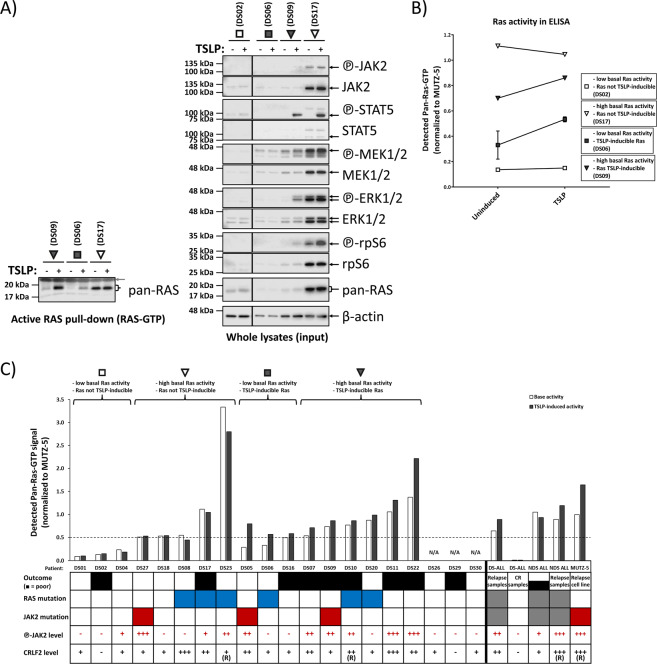
70% of primary DS-ALL presentation samples show activated and/or TSLP-inducible RAS, regardless of mutation-status. Primary presentation samples of DS-ALL patients were cultured for 2 days (detailed in Supplementary Fig. S5A-legend) and then induced for 10 min with 20 ng/mL TSLP (or uninduced) in serum-reduced medium. **a** Each lysate was split up for RAS-GTP pull-down assay (left blot) and for standard WB (right blot). Gray arrow shows the loading of the GST-RBD in the pull-down assay. **b** The RAS activity pattern in the patient samples from **a** was confirmed via ELISA measurement of RAS-activity in aliquots that were independently thawed and processed. **c** Overview of the ELISA-measured RAS activity for the DS-ALL cohort at diagnosis (not enough cell material was available for DS26, DS29, and DS30). The RAS-GTP pull-down ELISA was performed on lysates (100 ng/μL total-protein) from cells at minimum 75% viability. Brackets on top indicate the four RAS activity patterns presented in **a** and **b**. For visualization purposes only in this graph, basal RAS-activity over 0.5 (median of all patient samples) MUTZ-5 basal RAS activity was grouped as high RAS activity while an increase by at least 10% RAS-GTP in TSLP-stimulated samples over uninduced samples in ELISA was classed as TSLP-inducible RAS. For visualization, JAK2-phosphorylation levels measured in WB were categorized as –(negative) = 0.00–0.05; + = 0.05–0.50; ++ = 0.50–1.00; +++ = 1.00–2.00, and CRLF2 protein-levels were categorized as –(negative) = 0.00–0.05; + = 0.05–0.20; ++ = 0.20–0.50; +++ = 0.50–1.50. None of these arbitrary threshold-groupings were used in the clustering analysis (Fig. 5). Known CRLF2-rearrangements are marked (R). All values are normalized to those measured for uninduced MUTZ-5 cells processed in parallel to patient cells. Table boxes: Outcome of leukemia (white = good outcome, black = poor outcome), *RAS* mutations (=blue) or *JAK2* mutations (=red) (gray = unsequenced). For patient/sample groups other than DS-ALL-diagnosis (Non-DS (NDS) at presentation, DS complete remission (CR), and DS/NDS at relapse) only averages are shown. For an overview of the WB data and analyzed protein expression/phosphorylation of all individual samples, see Supplementary Fig. S5.

The MUTZ-5 ALL cells used in the analysis so far share the increased CRLF2 expression and mutated JAK2 with approximately a third of DS-ALL patients [20], which also have no mutations in RAS genes. We therefore analyzed primary cells from presentation samples (at primary diagnosis) of DS-ALL in RAS pull-down WB and ELISA assay measurements (+/- TSLP stimulation). The analyses were performed blinded to the mutation profile of the patient material and distinct DS-ALL patient profiles for RAS-activity and TSLP-inducibility of RAS were observed in WB (Fig. 4a) and confirmed by ELISA (Fig. 4b). As we see examples of RAS-mutated, wtRAS, or JAK2-mutated DS-ALL in each of the profile types (Fig. 4c), with exception of low-RAS and non-inducible type, we can conclude that activity levels and TSLP-inducibility of RAS cannot be predicted on the basis of DNA-sequencing (acquired mutations) patterns.

The most important conclusion of this analysis is that RAS is active/inducible in 14/20 (70%) of primary DS-ALL samples analyzed, 8 of which had no RAS mutations, but 75% of those had either mutated or hyperphosphorylated JAK2 (Fig. 4c). This means that either the RAS mutation, or the combination of high CRLF2 and hyperphosphorylated JAK2 (including mutated JAK2) can explain the mechanism for high RAS activity in 12/14 (86%) of DS-ALL with high RAS activity. Importantly, not a single wtRAS case with either mutated or hyperphosphorylated JAK2 was seen that lacked activated RAS protein (Fig. 4c), suggesting that RAS activation is an obligatory consequence in wtRAS DS-ALL cases with mutated or hyperphosphorylated JAK2.

### RAS activity and its TSLP inducibility correlate with outcome in DS-ALL patients

**Fig. 5:**
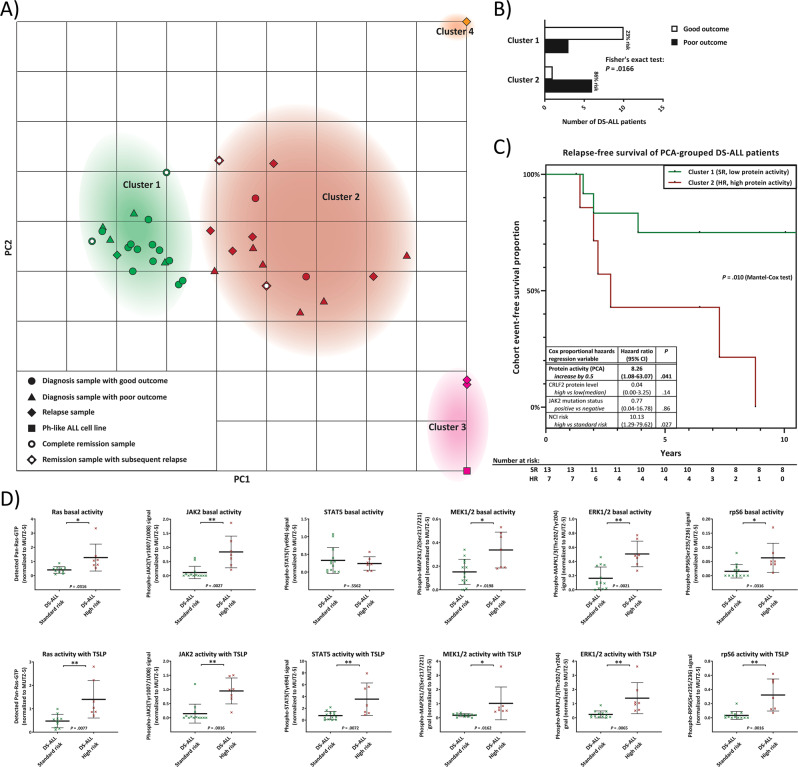
Sub-stratification of DS-ALL patients based on primary cells: RAS-activation and downstream signaling in relation to standard-therapy outcomes. **a** A PCA was performed on the quantified data of Fig. 4 (data was given as continuous variables; no cutoffs nor pre-groupings were used) for the DS-ALL cohort at diagnosis, and (where available) at remission, and relapse, as well as presentation and relapse samples from Non-DS ALL patients. Top view of the PCA-mapping for all six analyzed protein-activities (basal and TSLP-induced) as well as CRLF2-protein expression of all samples along the calculated principal components (see also Supplementary Fig. S6A). *K*-means unsupervised clustering (with *k* set to 4 to achieve minimal class-class deviation, Supplementary Fig. S6B) grouped samples into clusters 1–4 (listed in Supplementary Fig. S6C). **b** PCA Clusters 1 and 2 contain all samples of the DS-ALL diagnosis cohort and were analyzed according to their outcome: A Fisher’s exact test determined the *P* value between the number of good and poor outcomes between the two clusters (bar graph). **c** Kaplan–Meier curves of cluster 1 (SR standard risk) and cluster 2 (HR high risk) DS-ALL patients. Table shows a Cox proportional-hazards model for protein activity score (PCA-derived principal component from all quantified protein activities at basal and TSLP-induced level) together with CRLF2-protein expression level (for CRLF2+ samples), NCI risk groups (SR: age at diagnosis 1–10 years and WBC < 50.000/µL; HR = children age >10 years and/or WBC > 50.000/µL; or unknown), and presence of activating JAK2-mutations. Reverse Kaplan–Meier median follow-up for *N* = 20 DS-ALL was 18.4 years. Patient numbers at risk for each year are given in the table. **d** The means of all analyzed basal or TSLP-induced protein activities are compared between the SR group (DS-ALL patients in PCA cluster 1) and the HR group (DS-ALL patients in PCA cluster 2). All error bars are SD; *P* values were calculated using Student’s *T* test and are adjusted with a Bonferroni-correction for sequential multiple comparison.

Data from primary cells analysis from *n* = 20 presentation samples of DS-ALL for the RAS/MAPK, PI3K/mTOR, and JAK/STAT pathway activity profiles using WB (Supplementary Figs. S5A and S5B), as well as ELISA for activated RAS-pull-down (Fig. 4c) were integrated with the similar data we obtained using *n* = 7 DS-ALL relapse and *n* = 4 DS-ALL remission samples, as well as *n* = 4 non-DS ALL presentation samples and *n* = 2 non-DS relapse samples. We performed a PCA using all of these integrated data on *N* = 37 samples from *n* = 31 individual patients, in parallel to the same readouts from the MUTZ-5 Ph-like ALL reference cell line, and the PCA result was mapped onto a coordinate system (Fig. 5a) using the three principal components (PC1-3, Supplementary Fig. S6A). Unsupervised *k*-means clustering grouped ALL samples into Clusters 1–4 (Fig. 5a). This analysis grouped almost all presentation and remission samples of 9-year event-free survival patients (good outcome) together into Cluster 1 (green symbols). In contrast, out of 15 samples grouped into Cluster 2 (red symbols), 13 samples (87%) were from patients with death or subsequent relapse as outcome. Clusters 3 and 4, further along the PC1-axis, consisted of a small number of exclusively relapse samples. Using an independent mathematical approach, unsupervised hierarchical clustering of the 20 DS-ALL presentation samples (Supplementary Fig. S6E) grouped 90% of the samples into the same groups as the PCA-mapping. The clustering revealed that presentation samples from Cluster 1 correlated with good outcome for DS-ALL patients while DS-ALL patients grouped into Cluster 2 showed a significantly increased risk of relapse (Fig. 5b, c). The PCA-derived protein activity score was independently predictive of outcome (*P* = 0.041) (Fig. 5c) when analyzed by a multivariate Cox regression model together with CRLF2 protein-expression, NCI-risk and JAK2-mutation status (or RAS-mutation status, not shown). Cluster 2 contains a subgroup of DS-ALL presentation samples that clustered closer to the MUTZ-5 sample (Cluster 3). Like MUTZ-5, these patient samples had high CRLF2-expression, high JAK2-phoshorylation, and all featured the pattern of high basal RAS-activity that is TSLP-inducible. An event-free survival analysis that treats these MUTZ-5-like DS-ALL samples as a separate subcluster indicated a lower median survival (Supplementary Fig. S6D) but a higher number of samples is required to reach statistical significance for such subgrouping.

We restricted the further analysis only to DS-ALL primary presentation samples, and quantitatively compared those that PCA grouped into Cluster 1 (PCA-predicted standard-risk (SR)) to those in Cluster 2 (PCA-predicted high-risk (HR)), for the basal activities (Fig. 5d top row) and TSLP-induced activities (Fig. 5d bottom row) of pan-RAS, JAK2, STAT5, MEK1/2, ERK1/2 and rpS6. We observed that basal and TSLP-induced activities of JAK2, ERK1/2 and rpS6 were significantly increased in HR-DS-ALL presentation samples compared to the SR group (within PCA-Cluster 1). For these proteins, correlation between risk and protein-activity/inducibility profile for our DS-ALL cohort, resembles previously reported findings for a different group of HR-ALL, the non-DS Ph-like ALL, grouped by the presence or absence of CRLF2 rearrangements [27]. Additionally, (and this has, to our knowledge, never been demonstrated for any ALL before), we also observed a significant increase in basal and TSLP-induced activity of both MEK1/2 and RAS in the HR-DS-ALL group, compared to the SR group. We also looked at protein expression levels and found RAS and rpS6 levels to correlate with the high-risk DS-ALL group (Supplementary Fig. S7A). This provided the rationale to look for differences in a larger non-DS ALL cohort (see Supplementary Results, Supplementary Figs. S7B, S8A, S8B).

Our data on primary patient material suggest the compulsory activation of RAS whenever elevated CRLF2 is present in combination with either mutated or otherwise activated JAK2. This would eliminate the selective advantage gained by a RAS mutation, explaining the mutual exclusion, however the underlying molecular mechanism remains to be explained. We therefore sought to further characterize the molecular mechanism behind the wtRAS activation in these leukemic cells.

### TSLP activates RAS directly and independently of PI3K/mTOR pathway activation

**Fig. 6:**
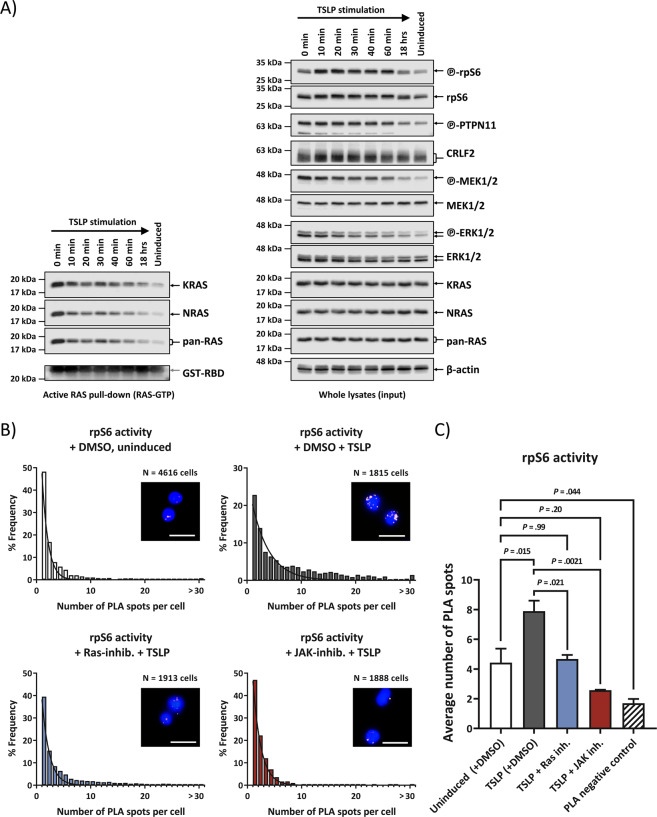
Direct wtRAS-activation can precede PI3K/mTOR-pathway activation and resulting PI3K-downstream signaling activity was blocked by RAS inhibitor. **a** Effect of TSLP induction over time. MUTZ-5 cells were incubated with 20 ng/mL human TSLP at 37 °C for the indicated time points (0 min to 18 h) before cell lysis. Due to the centrifugation step the TSLP can act for 5 min before lysis at timepoint 0. Each cell lysate was split up for RAS-GTP pull-down assay and WB. RAS-GTP pull-down elutions are on the left side while the right-hand side blots show whole cell lysates of the same samples. Antibody-targets are labeled on the right side of each image with black arrows indicating the respective protein band. **b** Activation of PI3K/mTOR downstream target rpS6 protein was monitored via PLA in high-throughput microscopy. MUTZ-5 cells were either not induced or induced with 20 ng/mL TSLP for 10 min. Where indicated, cells were pre-treated for 3 h with either DMSO (vehicle control), RAS inhibitor, or JAK inhibitor. Cells were fixed and permeabilized in a 96 well plate. After blocking, antibodies against phosphorylated rpS6 and total rpS6 were used in conjunction with PLA rabbit and mouse probes to allow specific readout of rpS6 activation in single cells in a high-throughput manner. Histograms show the distribution for a single experiment of the number of PLA spots in cells with at least 1 PLA spot (assay control is only shown in the bar graph). A minimum of 600 cells were analyzed per sample. Non-linear Gaussian fitting curves were plotted. Fluorescent microscope images show examples of PLA spots in MUTZ-5 cells for the respective treatment; white scale bars are 20 µm long. **c** The bar graph summarizes the average PLA spot counts of three independent experiments. Error bars are SD and *P* values were determined in one-way ANOVA and post-hoc Bonferroni multiple comparison.

The use of inhibitors on MUTZ-5 cells (Fig. 3a) suggested RAS activation to be independent from blocking of PI3K or JAK pathways. TSLP induction in high CRLF2-expressing and JAK2-mutated B-ALL is known to activate STAT5 and PI3K/mTOR pathways [27], and this insight is exploited in innovative new therapeutic approaches that are currently clinically trialed [6]. We therefore first confirmed that our experimental system can reproduce these same results in WB (Supplementary Fig. S4A). In addition, we designed a quantitative method (PLA) to measure rpS6-phoshporylation in individual cells (Fig. 6b). Similar to the TSLP-induction in MUTZ-5 cells, the Ba/F3 CRLF2 + JAK2_R683G_ cells also display an increased rpS6-phosphorylation in PLA compared to cells overexpressing only JAK2_R683G_ (Supplementary Fig. S3A).

Downstream effectors of the activated PI3K/mTOR pathway have been shown in some situations to cross-activate the downstream effectors of RAS-MAPK cascade, and vice versa [28, 29]. However, we observed that immediately upon addition of TSLP (0 min timepoint, Fig. 6a) the relative levels of activated pan-RAS, KRAS, NRAS, PTPN11, MEK1/2, and ERK1/2 were all higher than at any later timepoint, while in comparison, the activity onset of the PI3K/mTOR downstream target rpS6 was delayed (Fig. 6a). This makes it less likely, at least as the initial effect of TSLP, that the activation of MEK1/2 and ERK1/2 in such leukemic cells is caused by the cross-talk from the activated PI3K pathway. As PI3K can also be an effector of RAS [30], we used an alternative biochemical approach (PLA) by which we demonstrated the ability of a chemical inhibitor of RAS (Salisarib) to block the TSLP-induced rpS6-activating phosphorylation (Fig. 6b, c), at a concentration lower than required to block EIF4EBP1 activity via mTOR-complex destabilization [31]. PLA also detected a strong interaction between RAS and the RBD-containing PI3K-subunit p110α in these cells, which could be reduced using Rigosertib, a RAS-GTP mimetic that inhibits RAS by binding to the RBD of RAS-effectors (Supplementary Fig. S2D).

Our data therefore strongly suggest that direct, wtRAS activation can precede, and to a certain extent promote, the PI3K/mTOR pathway activation in TSLP-induced human ALL cells.

### CRLF2-signaling increases direct interaction between active PTPN11 and RAS

**Fig. 7:**
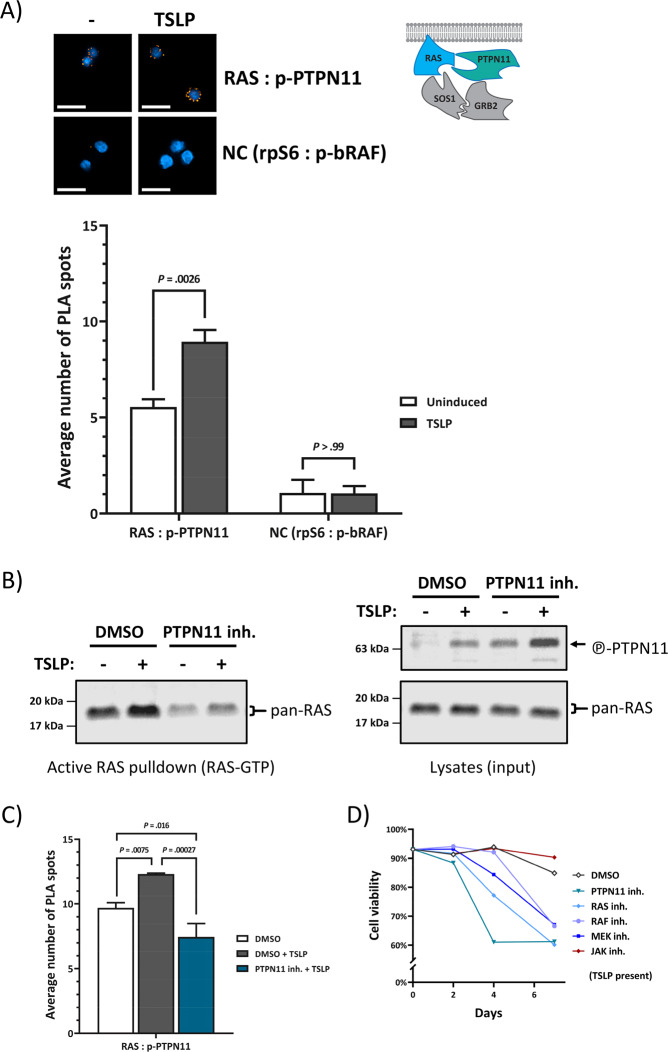
CRLF2-signaling induces direct interaction between activated PTPN11 and RAS, and PTPN11-activity is required for ALL cell growth. **a** Direct interaction between RAS and phosphorylated PTPN11 was monitored via PLA using high-content microscopy. Serum-starved MUTZ-5 cells were induced (or not) with 20 ng/mL TSLP for 10 min. Cells were fixed and permeabilized in a 96 well plate. Antibodies against phosphorylated PTPN11 and pan-RAS were used in conjunction with PLA-probes to allow the amplification and staining of interaction-specific PLA-spots. The negative control (NC) are two cytosolic proteins not expected to interact. Fluorescent-microscopy images show examples of PLA-spots (scale bars = 20 µm). At least 250 cells per well were analyzed using Operetta-CLS automated high-content microscopy platform. The bar-graph shows the averages of three independent experiments (each performed in triplicates). Error bars are SD and *P* values were determined in one-way ANOVA and post-hoc Bonferroni multiple comparison. **b** MUTZ-5 cells were pre-incubated with DMSO or 25 µM II-B08 (PTPN11 inhibitor) for 2 h and then stimulated or not with 20 ng/mL TSLP for 10 min before cell lysis. Each cell lysate was split up for RAS-GTP pull-down assay and for WB (whole-cell lysates). **c** MUTZ-5 cells were treated as in **b** before fixation. A PLA described in **a** was performed. **d** MUTZ-5 cells were seeded at 1.6 × 10^5^/mL density and cultured for 7 days with either 0.5% DMSO (vehicle control), 50 µM Salirasib (indirect pan-RAS inhibitor), 25 µM II-B08, 50 μM Vemurafenib (pan‐Raf inhibitor), 1 μM PD0325901 (MEK1/2-inhibitor), or 5 µM Ruxolitinib (JAK-inhibitor), in presence of 20 ng/mL TSLP. Percentage of viable cells was determined in an NC-250 automated cell counter.

While the PTPN11-inhibitor reduced wtRAS activity in MUTZ5- cells (Fig. 3c), the connection between PTPN11 and RAS in ALL as well as in CRLF2-signaling is unknown. Active PTPN11 is thought to dephosphorylate RAS to prime it for activation [32], and we found PTPN11 phosphorylation to be increased by induced CRLF2-signaling (Fig. 2a). Furthermore, PTPN11 is published to be in complex with JAK2 upon cytokine-induction in tumor cells [33]. In order to confirm that the mechanism of activating RAS in JAK2-mutated B-ALL cells is regulated via PTPN11, we designed a PLA assay that specifically detects the direct interaction between RAS and phosphorylated PTPN11 (Fig. 7a, c). Indeed, compared to the signal for two cytosolic proteins not expected to interact (PLA negative control (NC)), a strong PLA signal between RAS and p-PTPN11 was observed and this interaction almost doubled upon TSLP-induction (Fig. 7a). Of note, Ba/F3 cells cultured with IL-3, which activated RAS (Supplementary Fig. S1B) and JAK2-phosohorylation, also featured a higher level of RAS and p-PTPN11 interaction in PLA compared to unstimulated cells (Supplementary Fig. S3B). PLA assays also detected interactions between SOS1 and GRB2 in these leukemic cells, as well as other direct interactions involved in RAS activation, which also showed response to CRLF2-activation (RAS and SOS1; GRB2 and p-PTPN11) (Supplementary Fig. S2C). Remarkably, blocking PTPN11-activity via the PTPN11-inhibitor II-B08 reduced both endogenous, and TSLP-induced RAS activity in these cells (Fig. 7b). The PTPN11-inhibitor did not reduce the phosphorylation marker on PTPN11 itself (Fig. 7b) but disrupted the direct interaction between RAS and p-PTPN11, lowering it to levels below those in uninduced cells (Fig. 7c). Furthermore, a cytotoxic assay showed leukemic cell viability to be reduced by the PTPN11-inhibitor, similarly as with the RAS-inhibitor (Fig. 7d). Taken together, these results show that the mechanism of wtRAS activation by CRLF2 signaling depends on its direct interaction with catalytically active PTPN11.

### Primary DS-ALL patient biopsies from high-risk sub-cohorts have a potent response to RAS-inhibition in vitro as a distinguishing feature

**Fig. 8:**
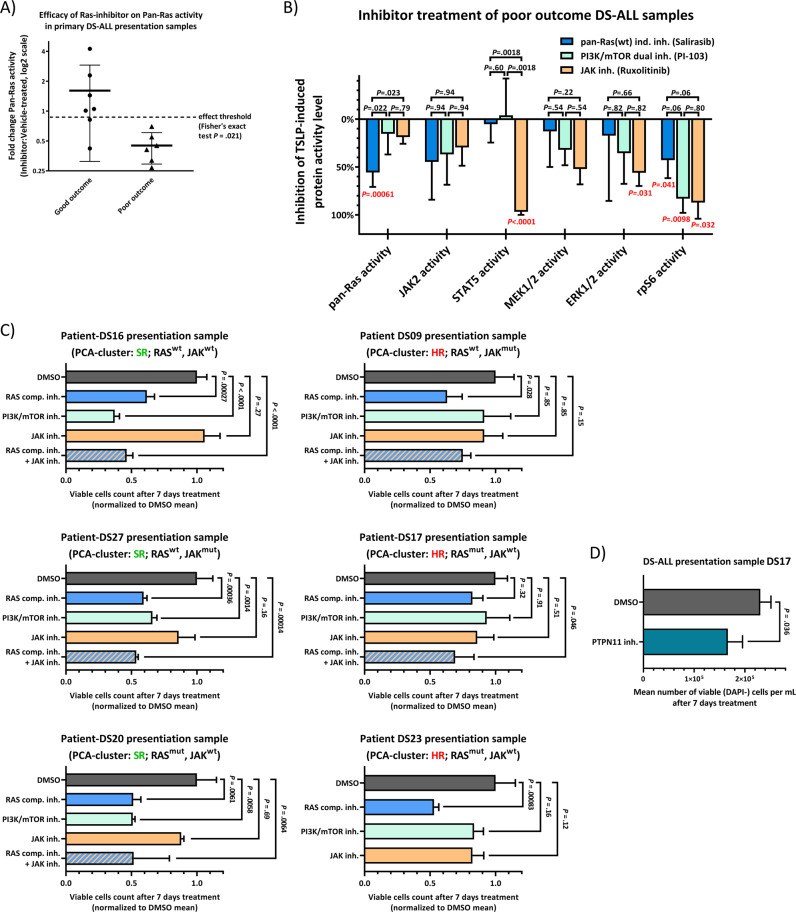
RAS-inhibitor blocks RAS-activity with greater efficiency in primary, poor outcome DS-ALL patient samples, prior to relapse. **a** Efficacy of RAS-inhibitor on ELISA-measured RAS-activity in DS-ALL, compared by outcome. Primary presentation DS-ALL samples were cultured for 2 days (detailed in Supplementary Fig. S5A-legend). Samples with sufficient cell count were treated with 0.5% DMSO (vehicle-control), or 50 µM Salirasib (indirect pan-RAS-inhibitor) for 3 h, and then induced for 10 min with 20 ng/mL TSLP in serum-reduced medium. Cells were lysed for RAS-GTP pull-down assay and whole-lysate WB (Supplementary Fig. S5A). Protein-activities of inhibitor-treated TSLP-induced samples were normalized to the activity-level of the respective vehicle-treated TSLP-induced samples. If inhibitor-treatment reduced the RAS-activity by over 10% compared to vehicle-control (dashed-line), the sample was tallied as successful RAS-blocking. A Fisher’s exact test was performed between the groups. Good outcome: *N* = 7 (3 *RAS*-mutations, 1 *JAK2*-mutation); poor outcome: *N* = 6 (1 *JAK2*-mutation). **b** Waterfall-plot shows the mean efficacies of 50 µM Salirasib, 10 µM PI-103 (PI3K/mTOR dual-inhibitor), or 5 µM Ruxolitinib (JAK-inhibitor) on pathway components (0% = no effect, 100% = full block of TSLP-induced protein-activation); tested on primary presentation samples from poor outcome DS-ALL patients in **a**. Error bars are SD; black *P* values were determined in one-way ANOVA with post-hoc Bonferroni multiple-comparison. The red *P* values (Bonferroni-corrected for sequential multiple-comparison) indicate if each inhibitor on average significantly reduced the respective protein-activity in these samples (only *P* < 0.05 shown; Supplementary Table S2 lists all *P* values). **c** Cell-toxicity effect of inhibitors in DS-ALL. Samples from six patients were cultured for 2 days like in **a** before seeding 8 × 10^5^ viable cells/mL in IMDM-complete medium (without IL-3/IL-7 but containing 20 ng/mL TSLP) together with 0.5% DMSO, 30 µM Rigosertib (non-ATP competitive RAS-GTP inhibitor), 10 µM PI-103, 5 µM Ruxolitinib, or Rigosertib&Ruxolitinib (DS23 cell count was insufficient). After 7 days, cell count and viability were measured (*N* = 3, means ± SD); vehicle-control cell numbers reduced to 1–4 × 10^5^/mL, 70–90% viability. *P* values were determined in one-way ANOVA with post-hoc Dunnett’s multiple-comparison (all treatments compared to DMSO-control). **e** 8 × 10^5^ viable cells/mL of patient-DS17 were handled like in **c** and treated with 0.5% DMSO, or 25 µM II-B08. After 7 days, cell count and viability were measured (*N* = 3, means ± SD); average vehicle-control viability: 74% (II-B08: 66%).

We used primary surplus clinical material in Fig. 5 from *n* = 31 patients. Out of these, we had enough primary diagnosis material for 13 patients (before any therapy) to measure the effects of RAS, PI3K, or JAK inhibitors on individual pathway activation status in the presence of TSLP. The efficacy of RAS inhibition on intracellular protein activity (expressed as panRAS activity ratio between inhibitor and vehicle treated samples) for primary presentation samples showed a significant difference (*P* = .021 by Fisher’s exact test) between the good outcome (*n* = 7) and poor outcome DS-ALL groups (*n* = 6) (Fig. 8a). Also, samples in which RAS can be further activated by TSLP were more sensitive to RAS inhibitor treatment (Supplementary Fig. S9). For all poor outcome DS-ALL primary presentation samples, inhibitions of individual pathway effector activities via the RAS, PI3K, or JAK inhibitors were visualized as inverted bar graphs ranging from 0% (no inhibition) to 100% (complete inhibition) (Fig. 8b). As indicated by red *P* values, PI3K inhibitor significantly inhibited rpS6 phosphorylation, whereas JAK inhibitor significantly inhibited ERK, rpS6, and STAT5 phosphorylation. Notably, these inhibitors did not have any significant effect on RAS activity, reproducing the result obtained for the MUTZ-5 Ph-like ALL cell line (Fig. 3a). Only the RAS inhibitor was able to significantly block RAS activation in poor outcome DS-ALLs (Fig. 8b), in addition to blocking rpS6 phosphorylation, as likewise shown for the MUTZ-5 cells (Figs. 6b and 3b). This suggests that only RAS-inhibitor action is capable of efficiently blocking RAS activation in cells from both Ph-like/non-DS and DS-ALL poor outcome patient samples at the point of first clinical presentation, irrespective of the presence of RAS mutation. In contrast, JAK and PI3K inhibitor treatments alone did not significantly impact RAS activity in these samples (Fig. 8b).

In order to better understand the physiological/therapeutic relevance, we measured the effect of RAS-inhibition on cell viability in six DS-ALL primary presentation samples (Fig. 8c). The competitive RAS-inhibitor Rigosertib was chosen over Salisarib as Rigosertib shows more potential in current clinical trials and still can disrupt both wtRAS and mutant-RAS signaling activity. Remarkably, after 7 days the RAS-inhibitor treatment had significantly reduced the viable cell count (almost halved compared to vehicle-control) in almost all six samples, independent of RAS-mutation status (Fig. 8c). Only in one patient sample (DS17) the RAS inhibitor needed a combinatorial-treatment (adding JAK-inhibitor) to achieve a similar reduction of viable cells. Cells of this patient treated separately with PTPN11-inhibitor resulted in a significantly reduced viable cell count (Fig. 8d).

The JAK-inhibitor alone showed no statistically significant effect in any of the DS-ALL samples, similar to what was observed for MUTZ-5 (Fig. 3a). Interestingly, while the PI3K/mTOR-inhibitor and the RAS-inhibitor both showed effective reduction of viable cells in all three samples (DS16, DS20, DS27) that were clustered by PCA as SR (Fig. 5), only RAS-inhibition was able to reduce the viable cell count in the samples grouped by the PCA (Fig. 5) as HR (DS09, DS17, DS23) (Fig. 8c).

These results suggest a paradigm shift in precision-therapy approach, by identifying HR sub-groups that are unlikely to respond to PI3K- or JAK-inhibitors alone and require direct RAS-inhibition. Importantly, the data confirm the notion that wtRAS-inhibitors could provide a uniform treatment for both mutated RAS and activated wtRAS cases (encompassing up to 80% of DS-ALL).

## Discussion

Both DS-ALL and Ph-like ALL share CRLF2-rearrangements and various kinase-activating alterations as potential targets for individualized therapy using specific kinase-inhibitors [8, 34]. This lead to the use of phosphorylation patterns of individual kinase signaling cascades as informative biomarkers for combinatorial therapy design [6, 16, 35].

In DS-ALL, recent studies of sub-clonal and single-cell evolution of changes in leukemic ALL blasts have identified signaling activators (CRFL2-rearrangements, JAK2 mutations, RAS-MAPK mutations and iAMP21) as frequent events in primary and relapsed leukemic blasts [20, 21, 36]. In particular, JAK2 and RAS mutations were found to be both acquired and lost in relapse samples in a mutually exclusive manner [20, 21]. This emphasizes the need for individualized combined-therapy approaches that have a better chance of preventing the selection of sub-clones driven by a different signaling. Our data show that elevation in CRLF2 levels combined with JAK2 activation are sufficient to activate wtRAS, and that TSLP has the potential to induce the wtRAS activity, independently of the PI3K/mTOR activity. This has implications on the choice of the combinatorial therapy design. Remarkably, our combined data from exome sequencing [20], and primary ALL cell protein signaling (presented in this study), suggest that up to 65–82% of DS-ALL cases have highly activated RAS, either constitutively, or upon TSLP induction, regardless of their mutation profiles. 12 of 14 cases with high RAS-activity featured either RAS mutations or high CRLF2/JAK2 signaling (including JAK2-mutations). The only two samples featuring high wtRAS activity in absence of high JAK2 phosphorylation levels might activate RAS via a different pathway yet to be uncovered for DS-ALL. A very recent novel patient-derived xenograft models for DS-ALL found *CBL-*mutant (wt*RAS*) cells to have as high ERK1/2-phosphorylation as *KRAS*-mutant cells [37]. Interestingly in the same study, the leukemia burden in both wt*RAS*(*JAK2-*mutant) and mutant-*RAS* xenograft models was reduced via MEK-inhibitor, representing a strongly corroborating evidence to some of the conclusions of our study.

Taking RAS activity and inducibility, integrated with other protein activation patterns, we performed a multivariate analysis clustering that identified SR and HR groups for DS-ALL and showed that protein activation pattern is independently predictive of outcome using multivariate Cox regression.

Ultimately, patient-specific inhibitor combinations based on analyzed pathway activities should be part of future precision medicine approaches for HR-ALL groups. Ph-like ALL patients are already being studied for the combined effects of PI3K/mTOR and JAK/STAT inhibitor treatment [6]. “Supplementary Discussion” contains an expanded discussion on RAS-inhibitor strategies.

Compared to RAS, mutations in *PTPN11* are less prevalent in DS-ALL but mutations in JAK2, RAS, and PTPN11 also appear to be mutually exclusive throughout different types of childhood ALL [20, 22, 38, 39]. Our data reveal that reducing RAS activity via inhibition of PTPN11 catalytical action may provide a functional alternative for ALL cells, while blocking the phosphorylation of PTPN11 via JAK inhibitors was not sufficient to prevent RAS activity, and concordantly with our mechanistic insight was also unable to block the direct interaction between PTPN11 and RAS. Our findings suggest that, depending on the patient’s protein activity profile, RAS inhibition (upstream, direct, or downstream) should be considered in combination with PI3K/mTOR and/or JAK/STAT inhibitors to further augment clinical treatment. In particular in DS-ALL, RAS/MAPK-inhibition might be applicable to most HR patients, as we show that specifically samples stratified by our PCA as HR seemed resistant to treatment with PI3K/mTOR or JAK inhibitors alone while only RAS-inhibition slashed the viable cell count in half. However, based on our data, the focus should not lie on targeting mutant-RAS alone but also the inhibition of overstimulated wtRAS pathway activity in absence of *RAS* mutations.

Childhood leukemia in DS is distinguished by a relatively specific pattern of acquired mutation changes, for both AML [40–45] and ALL [20, 21, 46], and the reasons for this are not fully explained. More generally, people with DS have an unusual epidemiological pattern of malignancy: increased incidence and mortality for childhood leukemias of all types, but much decreased childhood and adult solid tumors [47, 48]. Functional consequences of an increased dose of some chromosome 21 genes may play important roles [48], and this is discussed in greater detail in “Supplementary Discussion”. It will be important to unravel the mechanisms behind the actions of these chromosome 21 genes, as their specific inhibition may be an additional component to consider in combinatorial therapy approaches [37, 49]. This is highlighted by very frequent observations of extra copies of chromosome 21 as acquired changes in DS and non-DS ALL, both at diagnosis, and at relapse [20, 50].

In conclusion, our data show that activation of RAS protein is a common feature of up to 80% of DS-ALL, suggesting inhibition of overstimulated RAS pathway activity should be a unifying therapeutic strategy, even in the absence of *RAS* mutations. Importantly, our data indicate that patient pre-stratification for therapy optimization should assess RAS/MAPK protein activation status.

## Supplementary information

Supplementary material
