# Supplementary material for: RAS-protein activation but not mutation status is an outcome predictor and unifying therapeutic target for high-risk acute lymphoblastic leukemia
Source: Oncogene. 2020 Nov 27;40(4):746–62. doi: 10.1038/s41388-020-01567-7 (PMC7843419; doi:10.1038/s41388-020-01567-7)
Supplement: Supplementary file 1 — Supplementary material [file 41388_2020_1567_MOESM1_ESM.pdf]

Supplemental material for:

RAS-protein activation but not mutation status is an outcome predictor and unifying therapeutic target for high-risk acute lymphoblastic leukemia

David Koschut,[1] Debleena Ray,[1] Zhenhua Li,[2] Emanuela Giarin,[3] Jürgen Groet,[4] Ivan Alić,[1,5] Shirley Kow-Yin Kham,[2] Wee Joo Chng,[6] Hany Ariffin,[7] David M. Weinstock,[8] Allen Eng-Juh Yeoh,[2,6] Giuseppe Basso,[3,9] and Dean Nižetić[1,4]

[1]Lee Kong Chian School of Medicine, Nanyang Technological University, Singapore

[2]Department of Paediatrics, Yong Loo Lin School of Medicine, National University of Singapore, Singapore

[3]Department of Women's and Children's Health (SDB), Hematology-Oncology Laboratory, University of Padua, Italy

[4]The Blizard Institute, Barts and The London School of Medicine and Dentistry, Queen Mary University of London, United Kingdom

[5]Department of Anatomy, Histology and Embryology, Faculty of Veterinary Medicine, University of Zagreb, Croatia

[6]National University Cancer Institute, Singapore

[7]University of Malaya Medical Centre, University of Malaya, Malaysia

[8]Department of Medical Oncology, Dana-Farber Cancer Institute, Harvard Medical School, USA

[9]Italian Institute for Genomic Medicine, Italy

Materials included:

Supplementary Material and Methods

Supplementary Results

Supplementary Discussion (including additional references)

Supplementary tables: Supplementary-Tab.S1-S3

Supplementary figures (and legends): Supplementary-Fig.S1-S9

Supplementary Material and Methods

Antibodies, inhibitors, and cytokines

The primary antibodies against β-actin (Cat.#ab8227; WB (WB)1:10,000) and GRB2 (Cat.#ab86713; PLA 1:100) were purchased from Abcam (Cambridge, UK); the antibody against CRLF2 (Cat.#AF981; WB 1 μg/mL) was purchased from R&D Systems (Minneapolis, US). The primary antibodies against pan-RAS (Cat.#8832; WB1:200), phospho-bRAF (Cat.#2696; WB1:1,000, PLA1:100), JAK2 (Cat.#3230; WB1:900), HA-tag (Cat.#2367; WB1:1,000), MEK1/2 (Cat.#8727; WB1:1,000), phospho-MEK1/2 (Cat.#9154; WB1:1,000), RPS6 (Cat.#2317; PLA1:50, WB1:1,000) and phospho-STAT5 (Cat.#4322; WB1:1,000), phospho-JAK2 (Cat.#3771; WB1:1,000), ERK1/2 (Cat.#9102; WB1:1,000), phospho-ERK1/2 (Cat.#9101; WB1:1,500), GRB2 (Cat.#3972; WB1:1,500), phospho-RPS6 (Cat.#2211; PLA1:50, WB1:1,000), PI3Kp110α (Cat.#4249; PLA 1:100), SOS1 (Cat.#5890; PLA 1:100), PTPN11 (Cat.#3752; WB1:1,000, PLA1:100), phospho-PTPN11 (Cat.#3751; WB1:1,000, PLA1:100), and STAT5 (Cat.#9363; WB1:1,000) were purchased from Cell Signaling Technology (Danvers, US). The primary antibodies raised against KRAS (Cat.#sc-30; WB1:180), NRAS (Cat.#sc-31; WB1:160) and HRAS (WB1:170) were bought from Santa Cruz Biotechnology (Dallas, US). The primary antibodies used in immunofluorescence against pan-RAS (Cat.#MA1-012; IF1:100, PLA1:100) and bRAF (Cat.#PA5-14926; IF1:50, PLA1:50, WB1:1,000) were purchased from ThermoFisher Scientific (Waltham, US), as was SOS1 (Cat.#MA5-17234; PLA1:100).

Secondary HRP-conjugated antibodies against mouse (Cat.#ab97023; WB1:8,000), rabbit (Cat.#ab97051; WB1:9,000), or goat (Cat.#ab97100; WB1:7,000) IgG species were obtained from Abcam. The secondary fluorescent antibodies anti-mouse IgG Alexa Fluor 488 (Cat.#A11029) and anti-rabbit IgG Alexa Fluor 594 (Cat.#A11037) were purchased from ThermoFisher Scientific.

The small molecule inhibitors PI-103 (PI3K/mTOR-inh.; Cat.#S1038), Ruxolitinib (JAK-inh.; Cat.#S1378), Salirasib (RAS-inh.; Cat.#S7684), Rigosertib (RAS-signaling-inh.; Cat.# S1362), PD0325901 (MEK1/2-inh.; Cat.#: S7684), and Vemurafenib (RAF-inh.; Cat.#S1267) were purchased from Selleck Chemicals

(Houston, US). Additionally, the PTP inhibitor XXXI/II-B08 (PTPN11-inh.; Cat.#565852; EMD Millipore, Burlington, US) was purchased. All inhibitors were reconstituted in dimethyl sulfoxide (DMSO; Cat.#D2650; Sigma-Aldrich, St. Louis, US).

The cytokine used for Ba/F3 culturing was 10 ng/mL murine IL-3 (Cat.#31310-03-10; Gold Biotechnology, St Louis, US).

SDS-PAGE and WB

Protein lysates (see "RAS activity assays") were mixed with 4×Laemmli buffer (Cat.#161-0747; Bio-Rad Laboratories, Hercules, US) containing fresh 200 mM DL-Dithiothreitol (Cat.#3483-12-3; Sigma-Aldrich). For most samples 5 µg total protein could be loaded. SDS-PAGE with 11%-resolving/5%-stacking acrylamide gels, and WB on PVDF-membrane (Cat.#88518; ThermoFisher Scientific) were performed according to the standard protocol of the equipment-manufacturer (Bio-Rad Laboratories). Each PVDF-membrane piece (per antibody) was separately imaged via the auto-exposure function of the ChemiDoc-MP imaging system (Bio-Rad Laboratories). Supplementary-Fig.S5B legend describes the WB-signal quantification in detail. PVDF membranes were stripped from antibodies using the Restore WB-Stripping buffer (Cat.#21059; ThermoFisher Scientific).

Proximity ligation assay (PLA)

MUTZ-5 cells at 1×10^6 cells/mL density were either not induced or induced with 20 ng/mL TSLP for 10 min. Where indicated, cells were pre-treated with either DMSO (vehicle control), RAS inhibitor, or JAK inhibitor for 3 hrs. Cells were fixed in 4% PFA in a 96-well plate for 15 min during which the plate was centrifuged at 400×g. Cells were permeabilized with methanol at -20 °C for 5 min. After blocking with 5% FBS, primary antibodies were incubated over night at 4 °C. On the next day, rabbit and mouse probes from the Duolink In Situ Orange kit (Cat.#DUO92102; Sigma-Aldrich) or the Duolink flowPLA Orange kit (Cat.#DUO94003; Sigma-Aldrich) were used and the PLA was performed according to the manufacturer's protocol.

Operetta CLS high-content screening microscope (PerkinElmer, Waltham, US) were used to detect and count the fluorescent PLA spots in at least 600 single cells for each well and condition. Spot distribution histograms and non-linear Gaussian fitting curves were plotted in Prism v8.1 (GraphPad Software, San Diego, US).

Principal component analysis (PCA)

ELISA and quantified WB data for all 38 samples successfully analyzed for this study were fed into the multidimensional vectoral data visualization software ViDaExpert v1.2(1). The 13 variables included for the PCA calculations were: pan-RAS, JAK2, STAT5, MEK1/2, ERK1/2, and rpS6 activity levels in absence or presence of TSLP as well as CRLF2 protein expression. Eigenvector-based multivariate analysis was performed and the contributions of the principal components 1-3 (PC1-3) of each original variable were calculated (Supplementary-Fig.S6A). Data was transformed to a new 3D coordinate system using the projection of PC1-3. For the *k*-means clustering of the patient samples, *k* was set to 4 after identifying the smallest, significantly different class-class deviation for *k* (Supplementary-Fig.S6B). The resulting coordinate system was loaded into Adobe Illustrator software for presentation. The heatmap was generated in R-software 3.6.0 (The R Foundation, Vienna, AT) by performing unsupervised hierarchical Ward's clustering algorithm on the DS-ALL presentation samples (using the same variables of the PCA), with correlation coefficient as distance metric.

Statistical analysis

For all multiple comparison analysis, one-way ANOVA and post-hoc Bonferroni calculations were performed in Prism v8.1. When only two samples were compared, a two-tailed, unpaired student t-test was performed. For each series of experiments that are not independent, an additional Holm-Bonferroni correction was carried out to adjust the *P*-values for sequential multiple comparison. All replicate experiments were handled and measured independently.

Kaplan–Meier survival estimator plots and multivariate analysis using Cox proportional-hazards model were calculated in R-software 3.6.0, for details on the used factors see the respective figure legends. Variable/category candidates stated in the respective figures were included because they were either the respective analyzed protein/mutation/activity or are known general ALL prognostic markers.

Phospho-protein antibody-microarray

MUTZ-5 cells were serum-starved for 16 hrs at 1×10^6 cells/mL density before being induced with 20 ng/mL TSLP (or uninduced) for 10 min at 37 °C. 8×10^6 cells per condition were lysed in 110 µL Extraction Buffer from the microarray assay kit (Cat.#KAS02; FullMoon BioSystems, Sunnyvale, USA). The antibody-microarray assay was performed according to the manufacturer's protocol and the native, Cy3-labeled proteins were allowed to bind to the antibody-microarray slides (Cat.#PJS202; FullMoon BioSystems). The slides were scanned using the Operetta CLS microscope and the spots for the 202 individual antibodies were analyzed using Protein Array Analyzer software v1.1.c in ImageJ. Each signal was normalized to the Cy3-control present on each microarray slide. To exclude changes that are due to epitope-obstruction from bound protein partners, the signal for phosphorylation-site-specific antibodies was normalized to the signal from each corresponding total antibody raised against the same (unphosphorylated) peptide sequence. Background was set as the average signal from BSA-spots (empty).

Ba/F3 cells transduction

Ba/F3 cells were nucleofected using a Nucleofector 2b (Lonza, Basel, CH) using Cell Line Nucleofector Kit V (Lonza) according to the manufacturer's protocol. The plasmids used for Ba/F3 transfection were MSCVpuromycinR-hCRLF2 (human wt CRLF2) and MSCVneomycinR-hJAK2RG (human $JAK2_{R683G}$ mutant)(2). In order to select for stably transfected cells, MSCVpuromycinR-hCRLF2-transfected cells were cultured with 1 µg/mL puromycin (Cat.#P8833; Sigma-Aldrich), MSCVneomycinR-hJAK2RG-transfected cells were cultured with 1.8 mg/mL G418 (Cat.#108321-42-2; Sigma-Aldrich), and co-

transfected cells were cultured with both antibiotics together. Cells transfected with the plasmid pMax-EGFP (Lonza) were used as control.

DNA- and RNA-sequencing of primary patient material

Patient samples were either whole exome-sequenced previously(3) or amplicons of *JAK2*, *KRAS* and *NRAS* (in addition, *HRAS* of MUTZ-5 cells) were amplified from genomic DNA and sent for Sanger sequencing (1st BASE, Singapore, SG) in both orientations and screened for mutations in Mutation Surveyor v5.0.1 (Softgenetics, State College, US). Oligonucleotides (Supplementary-Tab.S3) were designed to cover known activating mutation hotspots.

RNA sequencing was performed for patients of the MS2003/2010 studies(4, 5) using TruSeq Stranded mRNA Library Prep kit (Illumina, San Diego, US); sequenced on HiSeq2000/2500 or NextSeq500 (Illumina). Reads were aligned to hg19-reference genome using Tophat2(6). The number of reads mapped to each gene were counted using featureCounts(7) and gene expression level was calculated as fragments per kilobase of transcript per million mapped reads (FPKM). GATK best practices was performed for variant calling on RNAseq(8). Data was submitted to the European Genome-phenome Archive (Accession-number EGAS00001001858).

Immunofluorescence

MUTZ-5 cells were fixated with 4% paraformaldehyde (PFA; Cat.#30525-89-4/158127; Sigma-Aldrich), permeabilized with methanol (Cat.#67-56-1; Sigma-Aldrich) at -20 °C and then stained with primary antibodies against either pan-RAS or bRAF. Secondary antibodies, labeled with either Alexa-488 (green) or Alexa-594 (red), specific to the respective primary antibody species were used to visualize the spatial organization of RAS (mostly at plasma membrane) and RAF (mostly in cytoplasma) proteins in a LSM800 inverted confocal microscope with Airyscan, 63×/NA1.20W objective (Carl Zeiss, Oberkochen, DE) at room temperature. Cell nuclei were stained with DAPI. Images were captured using ZEN software (Carl Zeiss) and processed in ImageJ.

Quantitative PCR

Quantitative PCR was performed using a StepOnePlus Real-Time PCR system (Applied Biosystems, Foster City, USA) with Power SYBR Green PCR master mix (Applied Biosystems) according to the standard manufacturer's protocol. The expression of human CRLF2 or human JAK2 was standardized against the expression of mouse GAPDH in each sample. The qPCR oligonucleotides are listed in Supplementary-Tab.S3.

Supplementary Results

(Expanded results description) Higher levels of RAS protein and mRNA correlate with poor outcome in DS-ALL and non-DS childhood ALL

The entire analysis shown so far was based on differences in activity of proteins targets. We also wanted to address protein expression levels in DS-ALL SR and HR groups. Based on individual protein expression levels (Supplementary-Fig.S7A), we found that overall levels of both RAS and rpS6 positively correlated with HR, but levels of CRLF2, STAT5, JAK2, MEK1/2, or ERK1/2 did not. In order to examine similar correlations in non-DS ALL, the material availability only permitted us to look for differences in mRNA transcript levels of the same genes within the whole transcriptome RNA-seq data of the MS2003/2010 cohort (N=346 non-DS ALL cases; median follow-up via reverse Kaplan-Meier = 7.64 years)(4, 5). *CRLF2* mRNA expression (Supplementary-Fig.S7B) and Cox regression hazard ratio (Supplementary-Fig.S8A) were significantly increased in the poor first event samples (n=47). We focused all further analysis on non-DS B-ALL samples with high-risk genetics: Moderate or high *CRLF2* expression (log2(FPKM+1)>0.7) and excluded ALL-subtypes that are considered to confer favorable outcomes (ETV6-RUNX1, Hyperdiploid, TCF3-PBX1, DUX4 and ZNF384). Within the resulting HR sub-cohort (n=91; median follow-up = 6.95 years) KRAS mRNA-expression was independently predictive of outcome (*P*=.030) when included in a multivariate analysis using Cox proportional hazards regression model together with Ph-like status, RAS mutation status, sex, and NCI risk (Supplementary-Fig.S8B). Of all analyzed genes, exclusively KRAS and JAK2 mRNA expression correlated with poor outcome (Supplementary-Fig.S7B). However, only high KRAS mRNA-levels were also significant in Kaplan–Meier survival estimator (Supplementary-Fig.S8B).

The combined data suggest that a large scale analysis on a non-DS ALL cohort is warranted for protein activation patterns of RAS, MEK1/2, and other pathway components activation readouts as this could potentially significantly inform the patient sub-stratification for outcome. This provides just a hint of a trend compatible with conclusions reached for the general role of RAS (irrespective of its mutational

status) as a biomarker in childhood ALL, but emphasizes the need to examine the protein-activation and inducibility parameters on a much larger cohort of non-DS ALL patients for a more accurate risk-prediction sub-stratification.

Supplementary Discussion

Case example: RAS activation explains the clonal evolution from presentation to relapse

DS-ALL patient DS09 highlights the need for an even deeper understanding of the driving mechanisms, and the need to block wtRAS activation as part of the combinatorial treatment design. Exome sequencing found at presentation a *JAK2*-mutation (and no *RAS*-mutation). These DS09 blast cells were highly TSLP-inducible for RAS activity, which could be blocked very efficiently using RAS-inhibitor Salisarib, but not using JAK or PI3K inhibitors in vitro. Concordantly in the experiment in Fig.8C, only RAS-inhibition using Rigosertib significantly reduced viable cell numbers in the DS09-sample. The same patient relapsed two years after standard chemotherapy regimen, and the relapse sample contained no JAK2 mutations anymore, but the main blast had gained an NRAS mutation (sequenced as sample 4-1036101-T2(3)). Interestingly, this relapse sample with constitutively activated NRAS shows no rpS6 activity any longer (with or without TSLP-induction) and TSLP could only induce moderate levels of STAT5 activity compared to the presentation sample (Supplementary-Fig.S5A).

(Expanded) RAS inhibition strategies

The importance of identifying RAS activity for the patient outcome relies on the availability of effective RAS-treatments. RAS has been commonly deemed to be 'undruggable', a term describing the lack of RAS inhibitors that perform well pharmacologically. The RAS inhibitor Salirasib, a Farnesyl Thiosalicylic Acid (FTS) acts non-direct, as a mimetic of RAS for binding to RAS-escort proteins which selectively disrupts the association of RAS to the plasma membrane. It showed no effect in clinical trials on solid cancers trials(9) and requires relatively high dosage. We chose this RAS inhibitor for being able to use a single inhibitor that can block RAS activity of all the main RAS isoforms and most importantly can also block wtRas activity independent of RAS mutations. A new generation of RAS inhibitors are on the horizon(10) such as mutant-specific inhibitors of KRAS(G12C)(11). However,

based on our data, the focus should not lie on targeting mutant-RAS alone but also the inhibition of overstimulated activity in absence of RAS mutations.

The RAS inhibitor Salirasib used in our study disrupts the spatiotemporal localization of active RAS but requires relatively high concentrations, thus rendering it ineffective in Phase II clinical trials(12). Newer RAS-inhibitors like the RAS-mimetic Rigosertib, which blocks the RBD in RAS-effectors, as seen for bRAF and p110αPI3K in our PLA analysis on MUTZ-5 cells, allowing the block of both wt and mutant RAS-activity. Rigosertib was effective in reducing the viable cell count in our DS-ALL samples, irrespective of mutation status, and is currently being evaluated in a Phase III study for MDS/AML(13).

Treatments involving the inhibition of activated RAS, independent of mutation status, that block the activation of multiple RAS-effector pathways, could help to cripple the cancer cells' ability to adapt.

(Expanded) Potential roles of chromosome 21 genes relevant to leukemogenesis

Increased propensity for early hematopoietic (both myeloid and lymphoid) cell fate can be influenced by trisomy of *RUNX1*(14, 15), whereas *ERG* trisomy is linked to skewing of cell fate towards megakaryocytic lineage (the most frequent AML form in DS)(16). Increased *HGMN1*-dose through trisomy 21 directly enhanced the early B-lymphocyte precursors, and could play a role as one of the initiating events in ALL leukemogenesis(17), whereas *CHAF1B* trisomy may increase the risk of AML(18). One of the most dose sensitive chromosome 21 genes known for multiple pathway de-regulations when copy number is increased, is *DYRK1A*. Increased *DYRK1A*-dose could promote both AML and ALL pathogenesis(19, 20). Interestingly, both *DYRK1A*, and another chromosome 21 gene *ITSN* also play a role in activating RAS, in specific cellular contexts(21, 22).

Supplementary references

1. Gorban AN, Pitenko, A., Zinovyev, A. ViDaExpert: user-friendly tool for non-linear visualization and analysis of multidimensional vectorial data. 2014(28 May 2019).

2. van Bodegom D, Zhong J, Kopp N, Dutta C, Kim MS, Bird L, et al. Differences in signaling through the B-cell leukemia oncoprotein CRLF2 in response to TSLP and through mutant JAK2. Blood. 2012;120(14):2853-63.

3. Nikolaev SI, Garieri M, Santoni F, Falconnet E, Ribaux P, Guipponi M, et al. Frequent cases of RAS-mutated Down syndrome acute lymphoblastic leukaemia lack JAK2 mutations. Nature communications. 2014;5:4654.

4. Yeoh AE, Ariffin H, Chai EL, Kwok CS, Chan YH, Ponnudurai K, et al. Minimal residual disease-guided treatment deintensification for children with acute lymphoblastic leukemia: results from the Malaysia-Singapore acute lymphoblastic leukemia 2003 study. J Clin Oncol. 2012;30(19):2384-92.

5. Yeoh AEJ, Lu Y, Chin WHN, Chiew EKH, Lim EH, Li Z, et al. Intensifying Treatment of Childhood B-Lymphoblastic Leukemia With IKZF1 Deletion Reduces Relapse and Improves Overall Survival: Results of Malaysia-Singapore ALL 2010 Study. J Clin Oncol. 2018;36(26):2726-35.

6. Kim D, Pertea G, Trapnell C, Pimentel H, Kelley R, Salzberg SL. TopHat2: accurate alignment of transcriptomes in the presence of insertions, deletions and gene fusions. Genome Biol. 2013;14(4):R36.

7. Liao Y, Smyth GK, Shi W. featureCounts: an efficient general purpose program for assigning sequence reads to genomic features. Bioinformatics. 2014;30(7):923-30.

8. Van der Auwera GA, Carneiro MO, Hartl C, Poplin R, Del Angel G, Levy-Moonshine A, et al. From FastQ data to high confidence variant calls: the Genome Analysis Toolkit best practices pipeline. Curr Protoc Bioinformatics. 2013;43:11 0 1-33.

9. Riely GJ, Johnson ML, Medina C, Rizvi NA, Miller VA, Kris MG, et al. A phase II trial of Salirasib in patients with lung adenocarcinomas with KRAS mutations. Journal of Thoracic Oncology: Official Publication of the International Association for the Study of Lung Cancer. 2011;6(8):1435-7.

10. Dang CV, Reddy EP, Shokat KM, Soucek L. Drugging the 'undruggable' cancer targets. Nat Rev Cancer. 2017;17(8):502-8.

11. Patricelli MP, Janes MR, Li LS, Hansen R, Peters U, Kessler LV, et al. Selective Inhibition of Oncogenic KRAS Output with Small Molecules Targeting the Inactive State. Cancer discovery. 2016;6(3):316-29.

12. Riely GJ, Johnson ML, Medina C, Rizvi NA, Miller VA, Kris MG, et al. A phase II trial of Salirasib in patients with lung adenocarcinomas with KRAS mutations. J Thorac Oncol. 2011;6(8):1435-7.

13. Navada SC, Fruchtman SM, Odchimar-Reissig R, Demakos EP, Petrone ME, Zbyszewski PS, et al. A phase 1/2 study of rigosertib in patients with myelodysplastic syndromes (MDS) and MDS progressed to acute myeloid leukemia. Leukemia research. 2018;64:10-6.

14. De Vita S, Canzonetta C, Mulligan C, Delom F, Groet J, Baldo C, et al. Trisomic dose of several chromosome 21 genes perturbs haematopoietic stem and progenitor cell differentiation in Down's syndrome. Oncogene. 2010;29(46):6102-14.

15. Lie ALM, Marinopoulou E, Lilly AJ, Challinor M, Patel R, Lancrin C, et al. Regulation of RUNX1 dosage is crucial for efficient blood formation from hemogenic endothelium. Development. 2018;145(5).

16. Salek-Ardakani S, Smooha G, de Boer J, Sebire NJ, Morrow M, Rainis L, et al. ERG is a megakaryocytic oncogene. Cancer Res. 2009;69(11):4665-73.

17. Lane AA, Chapuy B, Lin CY, Tivey T, Li H, Townsend EC, et al. Triplication of a 21q22 region contributes to B cell transformation through HMGN1 overexpression and loss of histone H3 Lys27 trimethylation. Nature genetics. 2014;46(6):618-23.

18. Volk A, Liang K, Suraneni P, Li X, Zhao J, Bulic M, et al. A CHAF1B-Dependent Molecular Switch in Hematopoiesis and Leukemia Pathogenesis. Cancer cell. 2018;34(5):707-23 e7.

19. Malinge S, Bliss-Moreau M, Kirsammer G, Diebold L, Chlon T, Gurbuxani S, et al. Increased dosage of the chromosome 21 ortholog Dyrk1a promotes megakaryoblastic leukemia in a murine model of Down syndrome. J Clin Invest. 2012;122(3):948-62.

20. Thompson BJ, Bhansali R, Diebold L, Cook DE, Stolzenburg L, Casagrande AS, et al. DYRK1A controls the transition from proliferation to quiescence during lymphoid development by destabilizing Cyclin D3. J Exp Med. 2015;212(6):953-70.

21. Kelly PA, Rahmani Z. DYRK1A enhances the mitogen-activated protein kinase cascade in PC12 cells by forming a complex with Ras, B-Raf, and MEK1. Molecular biology of the cell. 2005;16(8):3562-73.

22. Mohney RP, Das M, Bivona TG, Hanes R, Adams AG, Philips MR, et al. Intersectin activates Ras but stimulates transcription through an independent pathway involving JNK. J Biol Chem. 2003;278(47):47038-45.

23. Yoda A, Yoda Y, Chiaretti S, Bar-Natan M, Mani K, Rodig SJ, et al. Functional screening identifies CRLF2 in precursor B-cell acute lymphoblastic leukemia. Proc Natl Acad Sci U S A. 2010;107(1):252-7.

Supplementary-Tab.S1) Clinical and biological characteristics of the leukemia samples

DS-ALL Sample ID	Presentation / Relapse	BM/PB	AIEOP-BFM subclassification	Blasts %	WBC	Diagnosis	Karyotype	Gender	NCI	Poor outcome (death or relapse)	Sample ID in: https://doi-org.ezlibproxy1.ntu.edu.sg/10.1038/ncomms5654	
DS01	Presentation	BM	B-II	65.0	N/A	ALL	N.D.	M	N/A	N	4-23-T1	
DS02	Presentation	BM	B-II	90.0	122800	ALL	N.D.	M	HR	Y		
DS04	Presentation	BM	B-II	95.0	N/A	ALL	47,XY,+21c	M	SR	N	4-1030604-T1	
DS05	Presentation	BM	B-II	95.0	N/A	ALL	N.D.	M	HR	N		
DS06	Presentation	BM	B-III	91.6	323000	ALL	N.D.	F	HR	N	4-37-T1	
DS07	Presentation	BM	B-II	87.0	86400	ALL	N.D.	F	HR	Y		
DS08	Presentation	BM	B-II	85.0	N/A	ALL	N.D.	F	SR	N		
DS09	Presentation	BM	B-II	70.0	18780	ALL	47,XY,+21c	M	SR	Y	4-1036101-T1	
DS10	Presentation	BM	B-II	62.0	35400	ALL	47,XY,+21c	M	HR	Y	4-1036272-T1	
DS11	Presentation	BM	B-III	91.0	N/A	ALL	48,XY,+X,+21	M	SR	Y		
DS16	Presentation	BM	B-II	90.0	2400	ALL	N.D.	M	HR	Y	4-44-T1	
DS17	Presentation	BM	B-II	90.0	44600	ALL	47,XY,+21c[1	M	HR	Y	4-03-T1	
DS18	Presentation	BM	B-II	86.0	23530	ALL	N.D.	M	SR	N	4-02-T1	
DS20	Presentation	BM	B-II	80.0	11500	ALL	47,XX,t(8;14)	F	SR	N	4-29-T1	also Remission sample tested
DS22	Presentation	BM	B-II	97.0	N/A	ALL	N.D.	F	NA	Y		
DS23	Presentation	BM	B-II	88.0	27400	ALL	47,XX,+21c	F	SR	N	4-1044929-T1	
DS26	Presentation	BM	B-II	88.0	N/A	ALL	47,XX,+21c[1	F	N/A	N		
DS27	Presentation	N/A	N/A	N/A	N/A	ALL	N/A	M	N/A	N		also Remission sample tested
DS29	Presentation	BM	B-II	87.0	55000	ALL	47,XY,+21c[1	M	HR	Y		
DS30	Presentation	PB	B-II	82.0	206000	ALL	N.D.	F	HR	N		
DS09R	Relapse	BM	B-II	47.0	12200	ALL	47,XY,+21c	M			4-1036101-T2	
DS12R	Relapse	BM	B-II	79.0	17000	ALL	N.D.	M				
DS16R	Relapse	BM	B-II	90.0	N/A	ALL	N.D.	M			4-44-T2	
DS19R	Relapse	BM	B-II	88.0	221400	ALL	N.D.	M				
DS22R	Relapse	BM	B-III	87.0	46000	ALL	N.D.	F			4-29-T2	
DS28R	Relapse	BM	B-II	83.0	98400	ALL	N.D.	M				
DS29R	Relapse	BM	B-II	83.0	39800	ALL	N.D.	M				
DS24m	Remission	BM	N/A	N/A	N/A	N/A	N.D.	M		Y		
DS25m	Remission	BM	N/A	N/A	N/A	N/A	N.D.	F		Y		

Non-DS ALL Sample ID	Presentation / Relapse	BM/PB	AIEOP-BFM subclassification	Blasts %	WBC	Diagnosis	Karyotype	Gender	NCI	Poor outcome (death or relapse)
NDS03	Presentation	N/A	N/A	N/A	N/A	ALL	N/A	F	N/A	N
NDS04	Presentation	BM	B-II	93	97800	ALL	N.D.	M	HR	N
NDS05	Presentation	BM	B-II	82.8	56000	ALL	46,XX	F	HR	Y
NDS06	Presentation	BM	B-II	89	80000	ALL	46,XX	F	HR	N
NDS01R	Relapse	BM	B-II	44.1	NA	ALL	N.D.	F		
NDS02R	Relapse	BM	B-II	97	NA	ALL	N.D.	M		

Supplementary-Tab.S1) Clinical and biological characteristics of the leukemia samples used in this study and therapy outcomes.

Supplementary-Tab.S2) List of Bonferroni-corrected *P*-values

Fig.1B)

**Comparison:**	**Bonferroni p-value after one-way ANOVA:**
— vs. $hJAK2^{R683G}$	>0.99999
— vs. hCRLF2	>0.99999
— vs. $hJAK2^{R683G}$ + hCRLF2	0.00160
$hJAK2^{R683G}$ vs. hCRLF2	>0.99999
$hJAK2^{R683G}$ vs. $hJAK2^{R683G}$ + hCRLF2	0.00364
hCRLF2 vs. $hJAK2^{R683G}$ + hCRLF2	0.00347

Fig.2A)

**Comparison:**	**p-value:**	**Holm-Bonferroni corrected p value:**
JAK2 activity uninduced vs. TSLP	0.00043	0.00171
MEK1/2 activity uninduced vs. TSLP	0.00065	0.00195
Pan-Ras activity uninduced vs. TSLP	0.00308	0.00616
PTPN11 activity uninduced vs. TSLP	0.01846	0.01846

Fig.2D)

**Comparison:**	**p-value:**	**Holm-Bonferroni corrected p value:**
KRas activity uninduced vs. TSLP	0.00216	0.00648
NRas activity uninduced vs. TSLP	0.01905	0.03810
HRas activity uninduced vs. TSLP	0.06632	0.06632

Fig.2E)

**Comparison:**	**p-value:**	**Holm-Bonferroni corrected p value:**
AKT2 (Ⓟ-Ser474) unind. vs. TSLP	0.01657	0.05404
CDKN1A (Ⓟ-Thr145) unind. vs. TSLP	0.00002	0.00015
cRAF (Ⓟ-Ser296) unind. vs. TSLP	0.01546	0.05404
ELK1 (Ⓟ-Thr417) unind. vs. TSLP	0.00016	0.00097
GAB2 (Ⓟ-Ser159) unind. vs. TSLP	0.01351	0.05404
MYC (Ⓟ-Ser62) unind. vs. TSLP	0.01025	0.05124
PTPN6 (Ⓟ-Tyr536) unind. vs. TSLP	0.01570	0.05404

Fig.6B)

**Comparison:**	**Bonferroni p-value after one-way ANOVA:**
Uninduced (+DMSO) vs. TSLP (+DMSO)	0.01572
Uninduced (+DMSO) vs. TSLP + Ras inh.	>0.99999
Uninduced (+DMSO) vs. TSLP + JAK inh.	0.20936
Uninduced (+DMSO) vs. PLA negative control	0.04445
TSLP (+DMSO) vs. TSLP + Ras inh.	0.02177
TSLP (+DMSO) vs. TSLP + JAK inh.	0.00214
TSLP (+DMSO) vs. PLA negative control	0.00102
TSLP + Ras inh. vs. TSLP + JAK inh.	0.13163
TSLP + Ras inh. vs. PLA negative control	0.03079
TSLP + JAK inh. vs. PLA negative control	>0.99999

Fig.8C)

**Comparison (each mean compared to respective control mean (DMSO))**	**Dunnett p-value after one-way ANOVA:**
**DS17**	
DMSO vs. RAS comp. inh.	0.32889
DMSO vs. PI3K/mTOR inh.	0.91477
DMSO vs. JAK inh.	0.51257
DMSO vs. RAS comp. inh. + JAK inh.	0.04654
**DS16**	
DMSO vs. RAS comp. inh.	0.00027
DMSO vs. PI3K/mTOR inh.	<0.0001
DMSO vs. JAK inh.	0.72408
DMSO vs. RAS comp. inh. + JAK inh.	<0.0001
**DS20**	
DMSO vs. RAS comp. inh.	0.00615
DMSO vs. PI3K/mTOR inh.	0.00588
DMSO vs. JAK inh.	0.69698
DMSO vs. RAS comp. inh. + JAK inh.	0.00643
**DS27**	
DMSO vs. RAS comp. inh.	0.00036
DMSO vs. PI3K/mTOR inh.	0.00143
DMSO vs. JAK inh.	0.1684
DMSO vs. RAS comp. inh. + JAK inh.	0.00014
**DS09**	
DMSO vs. RAS comp. inh.	0.02829
DMSO vs. PI3K/mTOR inh.	0.85979
DMSO vs. JAK inh.	0.85979
DMSO vs. RAS comp. inh. + JAK inh.	0.15412
**DS23**	
DMSO vs. RAS comp. inh.	0.00083
DMSO vs. PI3K/mTOR inh.	0.1601
DMSO vs. JAK inh.	0.12471

Fig.3A)

**Comparison:**	**Bonferroni p-value after one-way ANOVA:**
DMSO uninduced vs. DMSO TSLP	>0.99999
DMSO uninduced vs. Ras inh. uninduced	<0.00001
DMSO uninduced vs. Ras inh. TSLP	<0.00001
DMSO uninduced vs. PI3K inh. uninduced	<0.00001
DMSO uninduced vs. PI3K inh. TSLP	<0.00001
DMSO uninduced vs. JAK inh. uninduced	>0.99999
DMSO uninduced vs. JAK inh. TSLP	0.33322
DMSO TSLP vs. Ras inh. uninduced	<0.00001
DMSO TSLP vs. Ras inh. TSLP	<0.00001
DMSO TSLP vs. PI3K inh. uninduced	<0.00001
DMSO TSLP vs. PI3K inh. TSLP	<0.00001
DMSO TSLP vs. JAK inh. uninduced	>0.99999
DMSO TSLP vs. JAK inh. TSLP	0.00589
Ras inh. uninduced vs. Ras inh. TSLP	>0.99999
Ras inh. uninduced vs. PI3K inh. uninduced	0.00111
Ras inh. uninduced vs. PI3K inh. TSLP	>0.99999
Ras inh. uninduced vs. JAK inh. uninduced	<0.00001
Ras inh. uninduced vs. JAK inh. TSLP	0.00006
Ras inh. TSLP vs. PI3K inh. uninduced	<0.00001
Ras inh. TSLP vs. PI3K inh. TSLP	0.31820
Ras inh. TSLP vs. JAK inh. uninduced	<0.00001
Ras inh. TSLP vs. JAK inh. TSLP	0.02665
PI3K inh. uninduced vs. PI3K inh. TSLP	0.00944
PI3K inh. uninduced vs. JAK inh. uninduced	<0.00001
PI3K inh. uninduced vs. JAK inh. TSLP	<0.00001
PI3K inh. TSLP vs. JAK inh. uninduced	<0.00001
PI3K inh. TSLP vs. JAK inh. TSLP	<0.00001
JAK inh. uninduced vs. JAK inh. TSLP	0.11829

Fig.7A

**Comparison:**	**p-value:**	**Holm-Bonferroni corrected p value:**
RAS : p-PTPN11	0.001306	0.002612
NC (rpS6 : p-bRAF) (uninduced vs TSLP)	0.956956	>0.999999

Fig.7C

**Comparison:**	**Bonferroni p-value after one-**
DMSO vs. DMSO + TSLP	0.00748
DMSO vs. PTPN11 inh. + TSLP	0.01592
DMSO + TSLP vs. PTPN11 inh. + TSLP	0.00027

Fig.5D)

**Comparison:**	**p-value:**	**Holm-Bonferroni corrected p value:**
Pan-Ras basal activity SR vs HR	0.01315	0.03165
JAK2 basal activity SR vs HR	0.00053	0.00265
STAT5 basal activity SR vs HR	0.55617	0.55617
MEK basal activity SR vs HR	0.00495	0.01980
ERK basal activity SR vs HR	0.00035	0.00209
S6 basal activity SR vs HR	0.01055	0.03165
Pan-Ras TSLP-induced activity SR vs HR	0.00383	0.00766
JAK2 TSLP-induced activity SR vs HR	0.00027	0.00161
STAT5 TSLP-induced activity SR vs HR	0.00241	0.00722
MEK TSLP-induced activity SR vs HR	0.01622	0.01622
ERK TSLP-induced activity SR vs HR	0.00163	0.00653
S6 TSLP-induced activity SR vs HR	0.00032	0.00162

Fig.8B)

**Comparison:**	**Bonferroni p-value after one-way ANOVA:**
**Pan-Ras activity**	
pan-Ras inh. vs. PI3K/mTOR dual inh.	0.023
pan-Ras inh. vs. JAK inh.	0.023
PI3K/mTOR dual inh. vs. JAK inh.	0.800
**JAK2 activity**	
pan-Ras inh. vs. PI3K/mTOR dual inh.	0.944
pan-Ras inh. vs. JAK inh.	0.944
PI3K/mTOR dual inh. vs. JAK inh.	0.944
**STAT5 activity**	
pan-Ras inh. vs. PI3K/mTOR dual inh.	0.606
pan-Ras inh. vs. JAK inh.	0.002
PI3K/mTOR dual inh. vs. JAK inh.	0.002
**MEK1/2 activity**	
pan-Ras inh. vs. PI3K/mTOR dual inh.	0.544
pan-Ras inh. vs. JAK inh.	0.228
PI3K/mTOR dual inh. vs. JAK inh.	0.544
**ERK1/2 activity**	
pan-Ras inh. vs. PI3K/mTOR dual inh.	0.824
pan-Ras inh. vs. JAK inh.	0.668
PI3K/mTOR dual inh. vs. JAK inh.	0.824
**rpS6 activity**	
pan-Ras inh. vs. PI3K/mTOR dual inh.	0.066
pan-Ras inh. vs. JAK inh.	0.066
PI3K/mTOR dual inh. vs. JAK inh.	0.808

Supplementary-Fig.S1C)

**Comparison:**	**Bonferroni p-value after one-**
**JAK2 mRNA levels:**	
— vs. $hJAK2^{R683G}$	0.00415
— vs. hCRLF2	>0.99999
— vs. $hJAK2^{R683G}$ + hCRLF2	0.01031
$hJAK2^{R683G}$ vs. hCRLF2	0.00413
$hJAK2^{R683G}$ vs. $hJAK2^{R683G}$ + hCRLF2	0.69131
hCRLF2 vs. $hJAK2^{R683G}$ + hCRLF2	0.01023

Supplementary-Fig.S1D)

**CRLF2 mRNA levels:**	**Bonferroni p-value after one-way ANOVA:**
— vs. $hJAK2^{R683G}$	>0.99999
— vs. hCRLF2	0.02509
— vs. $hJAK2^{R683G}$ + hCRLF2	0.00020
$hJAK2^{R683G}$ vs. hCRLF2	0.02514
$hJAK2^{R683G}$ vs. $hJAK2^{R683G}$ + hCRLF2	0.00020
hCRLF2 vs. $hJAK2^{R683G}$ + hCRLF2	0.00077

Supplementary-Fig.S1E)

**Comparison:**	**Bonferroni p-value after one-way ANOVA:**
**Day4:**	
— vs. $hJAK2^{R683G}$	>0.99999
— vs. hCRLF2	0.98323
— vs. $hJAK2^{R683G}$ + hCRLF2	0.00016
$hJAK2^{R683G}$ vs. hCRLF2	0.98323
$hJAK2^{R683G}$ vs. $hJAK2^{R683G}$ + hCRLF2	0.00016
hCRLF2 vs. $hJAK2^{R683G}$ + hCRLF2	0.00022

Supplementary-Fig.S2C,D,E)

**Comparison:**	**p-value:**	**Holm-Bonferroni corrected p value:**
GRB2 : phospho-PTPN11 (uninducedvsTSLP)	4.1E-06	0.000434405
pan-RAS : bRAF (TSLPvsRigo)	8.2E-05	0.006362897
pan-RAS : p110aPI3K (TSLPvsRigo)	0.00016	0.0118114
pan-RAS : phospho-PTPN11 (TSLPvsRigo)	0.10487	0.97912792
pan-RAS : phospho-PTPN11 (uninducedvsTSLP)	1.7E-07	2.19501E-05
pan-RAS : PTPN11 (TSLPvsRuxo)	7E-06	0.000709391
pan-RAS : SOS1 (uninducedvsTSLP)	0.00029	0.019530583
rpS6 : phospho-bRAF (TSLPvsRigo)	3.6E-06	0.000387867
rpS6 : phospho-bRAF (TSLPvsRuxo)	0.0004	0.025484468
rpS6 : phospho-bRAF (uninducedvsTSLP)	0.00148	0.070801112
rpS6 : phospho-rpS6 (TSLPvsRigo)	2E-05	0.001828029
rpS6 : phospho-rpS6 (TSLPvsRuxo)	9.9E-08	1.30439E-05
rpS6 : phospho-rpS6 (uninducedvsTSLP)	3.4E-07	4.28713E-05
SOS1 : GRB2 (uninducedvsTSLP)	0.00023	0.016345942

Supplementary-Fig.S4B)

**Comparison:**	**p-value:**	**Holm-Bonferroni corrected p value:**
STAT5 activity uninduced vs. TSLP	0.00006	0.00039
JAK2 activity uninduced vs. TSLP	0.00043	0.00256
MEK activity uninduced vs. TSLP	0.00065	0.00325
rpS6 activity uninduced vs. TSLP	0.00067	0.00325
Pan-Ras activity uninduced vs. TSLP	0.00308	0.01053
PTPN11 activity uninduced vs. TSLP	0.01846	0.03692
ERK activity uninduced vs. TSLP	0.02192	0.03692

Supplementary-Fig.S7A)

**Comparison:**	**p-value:**	**Holm-Bonferroni corrected p value:**
Pan-Ras protein expression SR vs. HR	0.00427	0.02564
JAK2 protein expression SR vs. HR	0.07137	0.28547
STAT5 protein expression SR vs. HR	0.14639	0.43916
MEK1/2 protein expression SR vs. HR	0.40022	0.80045
ERK1/2 protein expression SR vs. HR	0.04039	0.20196
rpS6 protein expression SR vs. HR	0.00034	0.00237
CRLF2 protein expression SR vs. HR	0.49585	0.80045

Supplementary-Fig.S7B)

**Comparison:**	**p-value:**	**Holm-Bonferroni corrected p value:**
KRAS mRNA good vs. poor outcome	0.02670	0.18693
JAK2 mRNA good vs. poor outcome	0.03945	0.23670
NRAS/KRAS mRNA good vs. poor outcome	0.04616	0.23670
STAT5B mRNA good vs. poor outcome	0.10881	0.43525
RPS6 mRNA good vs. poor outcome	0.81850	>0.99999
MEK1/2 mRNA good vs. poor outcome	0.87458	>0.99999
ERK1/2 mRNA good vs. poor outcome	0.95096	>0.99999

**Comparison:**	**p-value:**	**Holm-Bonferroni**
STAT5 phosph. (JAK inh. vs. vehicle control)	0.00000	0.00001
Pan-Ras-GTP level (Ras inh. vs. vehicle control)	0.00004	0.00061
rpS6 phosph. (PI3K inh. vs. vehicle control)	0.00062	0.00988
ERK1/2 phosph. (JAK inh. vs. vehicle control)	0.00213	0.03193
rpS6 phosph. (JAK inh. vs. vehicle control)	0.00231	0.03231
rpS6 phosph. (Ras inh. vs. vehicle control)	0.00320	0.04154
MEK1/2 phosph. (JAK inh. vs. vehicle control)	0.00500	0.06004
MEK1/2 phosph. (PI3K inh. vs. vehicle control)	0.00737	0.08112
Pan-Ras-GTP level (JAK inh. vs. vehicle control)	0.01001	0.10007
JAK2 phosph. (Ras inh. vs. vehicle control)	0.03552	0.31971
JAK2 phosph. (PI3K inh. vs. vehicle control)	0.05956	0.47652
JAK2 phosph. (JAK inh. vs. vehicle control)	0.06092	0.47652
ERK1/2 phosph. (PI3K inh. vs. vehicle control)	0.06835	0.47652
Pan-Ras-GTP level (PI3K inh. vs. vehicle control)	0.28436	>0.99999
MEK1/2 phosph. (Ras inh. vs. vehicle control)	0.46249	>0.99999
STAT5 phosph. (Ras inh. vs. vehicle control)	0.56159	>0.99999
ERK1/2 phosph. (Ras inh. vs. vehicle control)	0.63041	>0.99999
STAT5 phosph. (PI3K inh. vs. vehicle control)	0.84173	>0.99999

Supplementary-Tab.S2) List of Bonferroni-adjusted or Holm-Bonferroni-corrected *P*-values from all statistical tests engaging in multiple comparisons in this article. Original *P*-values before Holm-Bonferroni-correction are also listed; the P-values appearing in figures and supplementary figures are highlighted red. See Materials and Methods for more details about the statistical methods used.

Supplementary-Tab.S3) List of oligonucleotides

human JAK2 sequencing:

Name:	Sequence:
Fwd-JAK2amp1,Seq1	CCACTCTTGCTCTCTCTCACTTT
Fwd-JAK2amp2,Seq2	CCATCCAGAAACACAAACCATGT
Fwd-JAK2amp3,Seq3	CGCCTGTATTCCCAGCTACT
Fwd-JAK2,Seq4	GGAAGTATTTGGCTAGTTGGT
Rev-JAK2amp3,rSeq1	CCTAATGTCTACTTCAACACGGT
Rev-JAK2,rSeq2	GGCAGCTTACCAGCACTGTA
Rev-JAK2amp2,rSeq3	CCTTTACACCACTGCCCAAGTAA
Rev-JAK2amp1,rSeq4	CCTAGCTGTGATCCTGAAACTGA

human KRAS sequencing:

Name:	Sequence:
Fwd KRas amplicon 1	GCGTCGATGGAGGAGTTTGTA
Rev KRas amplicon 1	GCACAGAGAGTGAACATCATGGA
Fwd KRas amplicon 2	CCACCAGCAATGCACAAAGATT
Rev KRas amplicon 2	CCAAAGCCAAAAGCAGTACCATG
Fwd KRas Seq3	GGTGTAGTGGAAACTAGGAATT
Rev KRas Seq4r	GCCACTGTTTATCCAATCCAA
Fwd KRas amplicon 3A	CCAGTTTCTTGACTCACCTTTGAG
Rev KRas amplicon 3A	GGGATAAGAAAGTGCTGTGCTG
Fwd KRas amplicon 3B	GCCTGAAGAGAAACATAAAGAATCC
Rev KRas amplicon 3B	GGGAATACTGGCACTTAGAGGAA

human HRAS sequencing:

Name:	Sequence:
Fwd HRas amplicon	GCTGTGGGTTTGCCCTTCA
Rev HRas amplicon	CCATGTCCTGAGCTTGTGCTG
Fwd HRas Seq2	GCCATCAACAACACCAAGTCTT
Rev HRas Seq3r	CCCATCAATGACCACCTGCTT
Fwd HRas Seq3	GGCACCTGTTGGTTCTGAGTCT
Rev HRas Seq2r	CCCACTAAGACTCAGAACCAACA

human NRAS sequencing:

Name:	Sequence:
Fwd NRas amplicon 1	GGGATTTCCATTGCTTAGGCT
Rev NRas amplicon 1	GGTTGGGAGAGATTCAGTTGCT
Fwd NRas amplicon 2	GGGACAAACCAGATAGGCAGAA
Rev NRas amplicon 2	CCCAAAGCACTGACATTCCAG
Fwd NRas amplicon 3	GGCTAATCTCAAACTCCTGGGTT
Rev NRas amplicon 3	GGTCATTGCCAGGAATAGGGTAT
Fwd NRas Seq4	CCCAGCCTGTTGTTAGGCATTT
Rev NRas Seq2r	GGCTGAGGCAGGAGAATCACTT

qPCR primers:

Name:	Sequence:
qRTPCR CRLF2 F	CCTTCTCCAGGAAGGTCACA
qRTPCR CRLLF2 R	GTCCCATTCCTGATGGAGAA
qRTPCR JAK2 F	CGGATAGATCACATAAAACTTCTGC
qRTPCR JAK2 R	TGCCAGATCCCTGTGGATA
mouse GAPDH qRT PCR fwd	GGTGCTGAGTATGTCGTGGA
mouse GAPDH qRT PCRrev	CGGAGATGATGACCCTTTTG

Supplementary-Tab.S3) List of oligonucleotides used to establish the mutation status of key genes involved in the analyzed pathways in the DS-ALL patients. Forward and reverse strand oligonucleotides were used to amplify genomic regions of interests (named Amp) in PCR and all primers were then used in Sanger sequencing on produced PCR amplicons. DNA of previously(3) whole-exome-sequenced samples was used as control for both wt and mutant sequence detection.

Supplementary Fig.S1

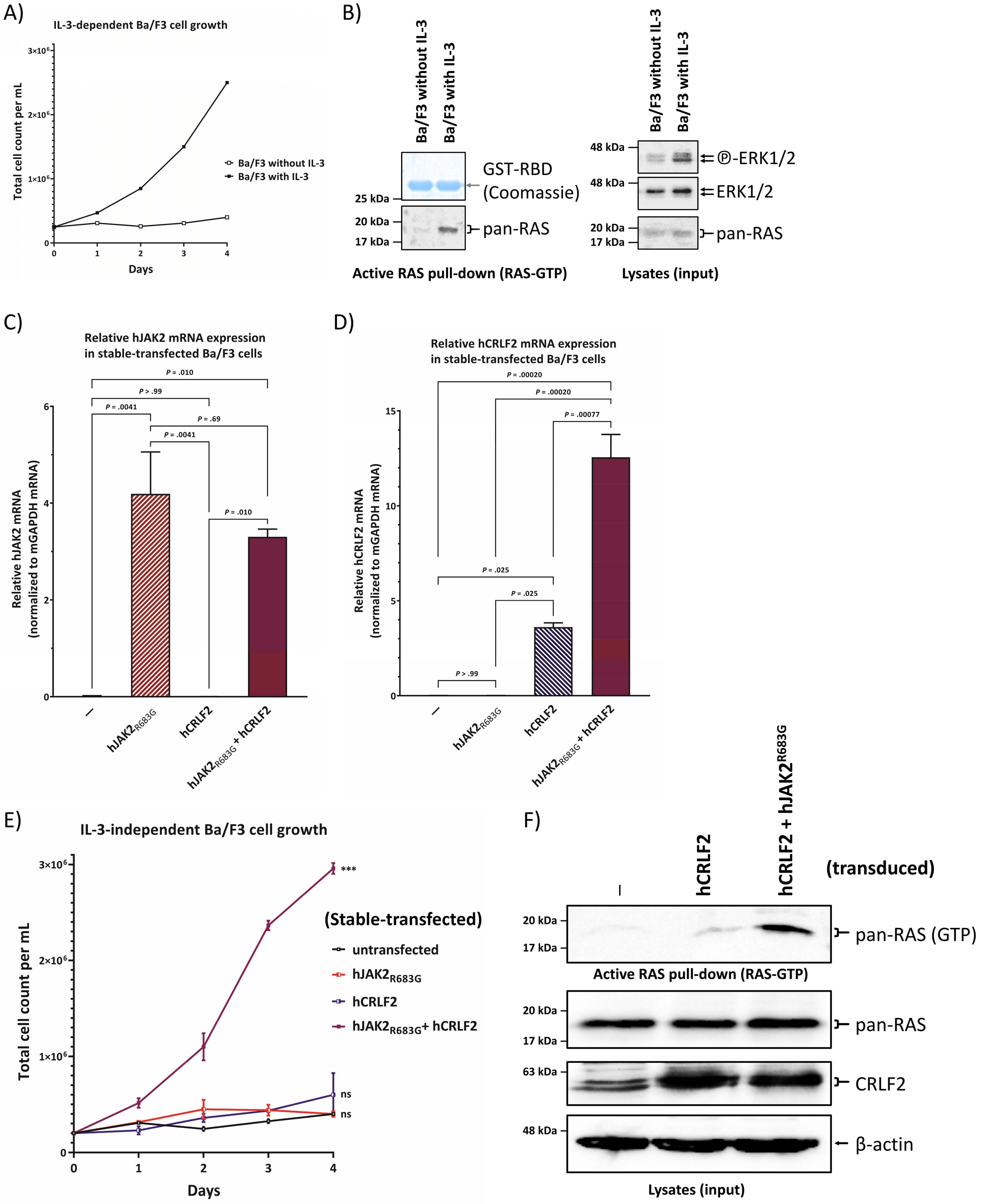

Supplementary-Fig.S1) IL-3 induces wtRAS activity and cell growth in murine pro B cells, but can be substituted by stable overexpression of constitutively active JAK2 and CRLF2. (A) Cell count of Ba/F3 cells (untransfected) over time. Cells were either cultured with 10 ng/mL IL-3 or without.
(B) Ba/F3 cells were cultured in 10 ng/mL IL-3. 17 hrs before lysis, the culture medium was changed to medium containing either 10 ng/mL IL-3 or no IL-3. A RAS-pull-down assay was performed. Lysates of pull-down and input were loaded on separate SDS-PAGE gels followed by Western blotting. Before Western blotting, the top part of the gel loaded with the RAS-GTP pull-down samples was stained with Coomassie dye to visualize the GST-RBD to ensure pull-down samples were loaded equally. To assess the total protein and phosphorylated protein amounts on the same PVDF-membrane, membranes were stripped and reprobed with new antibodies.
(C&D) Ba/F3 cells were stably transfected with hJAK2$_{R683G}$, hCRLF2, or both constructs together. Quantification of qPCR results for human JAK2 mRNA expression (A) and human CRLF2 mRNA expression (B) for the stably transfected Ba/F3 cell lines. All gene expression levels were normalized to murine GAPDH expression. Error bars are SD and *P*-values were determined in one-way ANOVA and post-hoc Bonferroni multiple comparison.
(E) Only the co-expression of human CRLF2 and a constitutively active JAK2$_{R683G}$ mutant construct enabled IL3-independent growth of Ba/F3 cells. The expression of either hCRLF2 or hJAK2$_{R683G}$ alone is not sufficient to promote cell growth over the course of 4 days. Data shown reproduced the experiments already published by Yoda et al.(23) to ensure that the cellular model recreated in our laboratory behaves the same and as evidence of the correlation with the state of RAS activation in these cells (Fig.1). Error bars are SD and *P*-values from a one-way ANOVA with post-hoc Bonferroni multiple comparison for the last timepoint are symbolized as asterisks (***: $P < 0.001$) or ns ($P > 0.05$).
(F) Ba/F3 cells were stably transduced (Lentiviral) with hCRLF2 alone, or followed by hJAK2$_{R683G}$. This second set of Ba/F3 lines was generated independently of the transfected lines used in (A-E) and Fig.1. All cells were then starved from IL-3 and cells were lysed. Each cell lysate was split up for analysis in RAS-GTP pull-down assay and for total proteins. An SDS-PAGE followed by WB was performed.

Supplementary Fig.S2

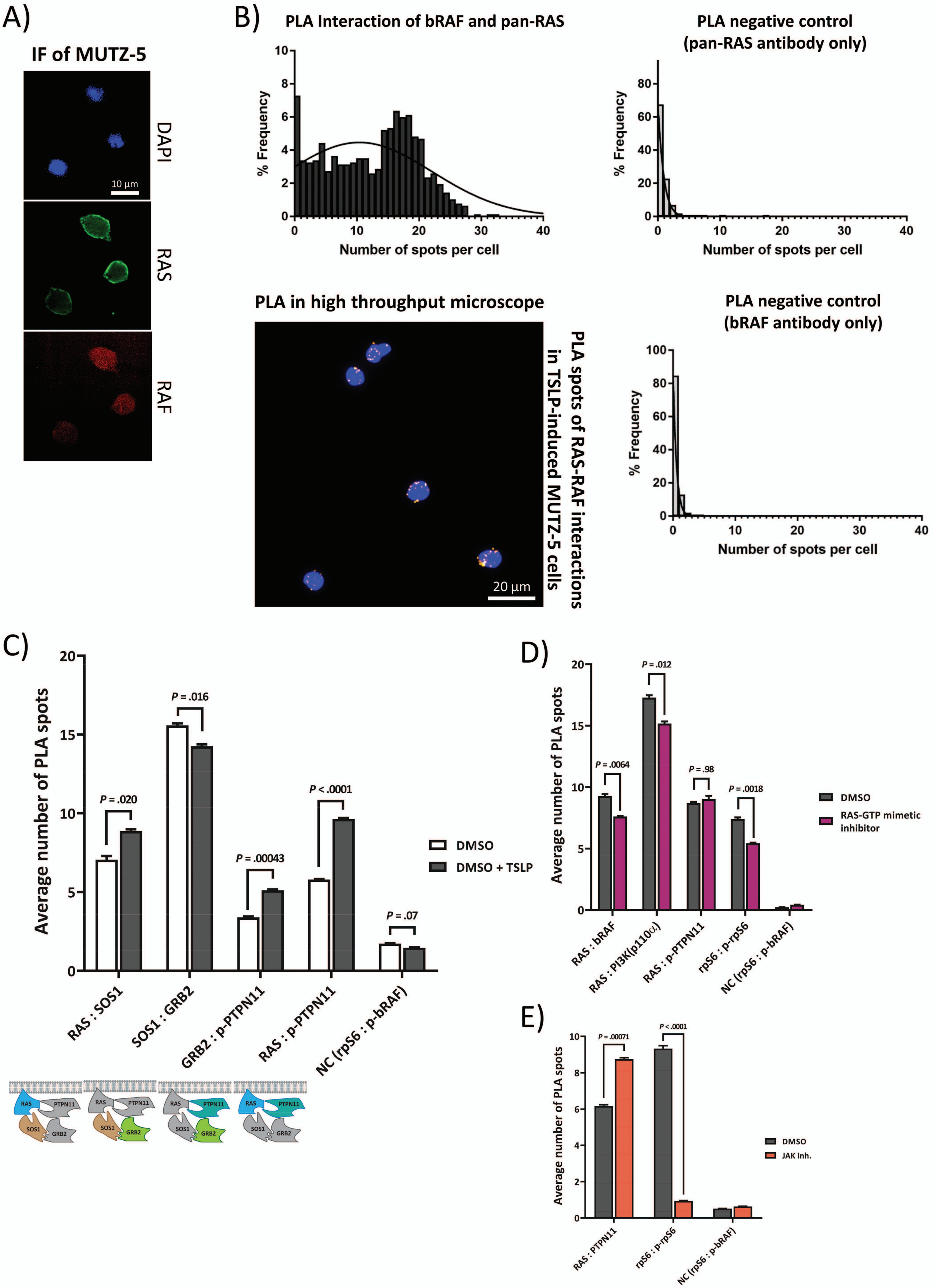

Supplementary-Fig.S2) CRLF2-signaling promotes direct protein-protein binding (quantitated by proximity ligation assays) of multiple components involved in RAS activation in the absence of RAS mutations in human Philadelphia-like (rearranged CRLF2, $JAK2_{R683G}$) B-ALL cells. (A) MUTZ-5 cells were PFA-fixed, permeabilized, and stained with DAPI. Primary antibodies against either pan-RAS or bRAF were used. Secondary antibodies specific to the respective primary antibody species labeled with either Alexa-488 (green) or Alexa-594 (red) were used to visualize the spatial organization of RAS (mostly plasma membrane) and RAF (mostly cytosolic) proteins in confocal microscopy.

(B) Direct interaction between individual RAS proteins and the MAPK pathway protein bRAF was monitored via proximity ligation assay (PLA) in high-throughput microscopy. MUTZ-5 cells were induced with 20 ng/mL TSLP for 5 min. After blocking, antibodies against Pan-RAS and bRAF were used in conjunction with PLA rabbit and mouse probes to allow specific readout of RAS binding to bRAF proteins in single cells in a high-throughput manner. Fluorescent microscope image shows the PLA spots in DAPI-stained cells. Histograms show the distribution of the number of spots in all cells, negative assay controls only received one of the antibodies (PLA for two non-interacting antigens showed higher levels of spots than the assay control, but remained at background levels, not shown). A minimum of 600 cells were analyzed per sample. Non-linear Gaussian fitting curves were plotted.

(C) Direct interaction between protein pairs involved in RAS activation (see cartoon below the graph) was monitored via PLA in high-throughput microscopy. MUTZ-5 cells were either not induced or induced with 20 ng/mL TSLP for 10 min. Cells were fixed and permeabilized in a 96 well plate. After blocking, the indicated antibody pairs were used in conjunction with PLA rabbit and mouse probes to allow the amplification and staining of interaction-specific PLA spots. The negative control (NC) samples used antibodies for two cytosolic proteins (rpS6 and phosphorylated bRAF) that were not expected to directly interact. At least 1900 cells per condition were analyzed in a high-throughput manner. The bar graph shows the averages of 3 technical replicates. Error bars are SD and *P*-values shown are Student's T-test after Bonferroni correction for sequential multiple comparison for all uninduced vs TSLP-induced pairs.

(D) Direct interaction between protein pairs involved in RAS activation and the binding between RAS to RAS effectors was monitored via PLA in high-throughput microscopy. MUTZ-5 cells treated with 20 ng/mL TSLP were pre-treated with either DMSO or the RAS-GTP mimetic inhibitor Rigosertib (30 µM) for 1.5 hrs. Cells were fixed and permeabilized in a 96 well plate. After blocking, the indicated antibody pairs were used in conjunction with PLA rabbit and mouse probes to allow the amplification and staining of interaction-specific PLA spots. The negative control (NC) samples used antibodies for two cytosolic proteins (rpS6 and phosphorylated bRAF) that were not expected to directly interact. At least 700 cells per condition were analyzed in a high-throughput manner. The bar graph shows the averages of 3 technical replicates. Error bars are SD and *P*-values shown are Student's T-test after Bonferroni correction for sequential multiple comparison for all DMSO vs Rigosertib pairs.

(E) Direct interaction between protein pairs between RAS and PTPN11 was measured via PLA in high-throughput microscopy. MUTZ-5 cells treated with 20 ng/mL TSLP were pre-treated with either DMSO or 5 µM Ruxolitinib (JAK inh.) for 1.5 hrs. Cells were handled like in (D). The condition using antibodies for rpS6 and phosphorylated rpS6 verified that the JAK inhibitor was effective in this experiment. At least 20,000 cells per condition were analyzed in a high-throughput manner. The bar graph shows the averages of 3 technical replicates. Error bars are SD and *P*-values shown are Student's T-test after Bonferroni correction for sequential multiple comparison for all DMSO vs Ruxolitinib pairs.

Supplementary Fig.S3

A)

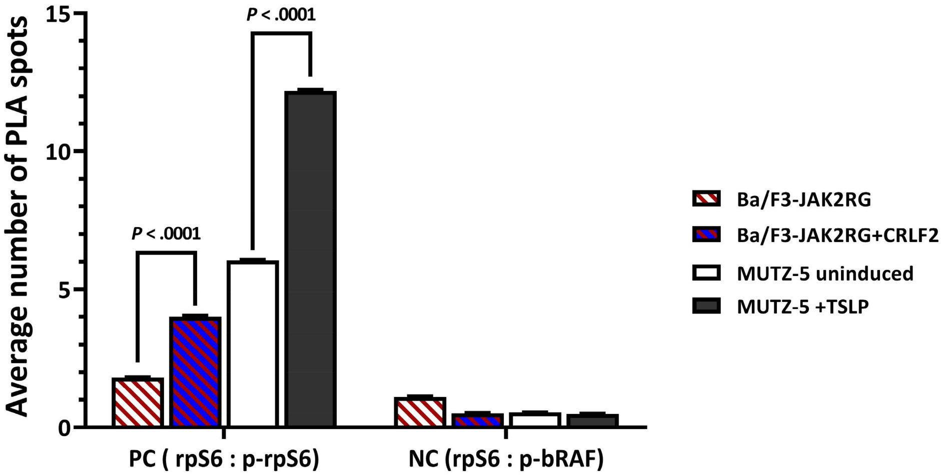

B)

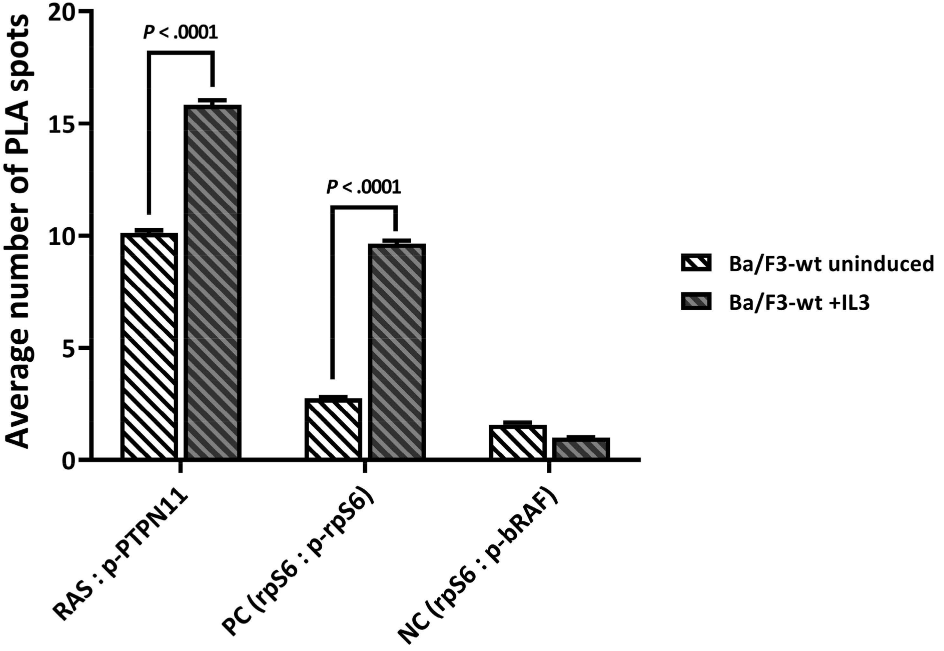

Supplementary-Fig.S3) PLA detects rpS6-phoshporylation and interaction between RAS and p-PTPN11 pattern in Ba/F3 lines comparable to MUTZ-5 cells.

(A) Activation of PI3K/mTOR downstream target rpS6 protein was monitored via PLA in high-throughput microscopy. Ba/F3 cell lines were IL-3 starved for 17 hrs. MUTZ-5 cells were either not induced or induced with 20 ng/mL TSLP for 10 min. Cells were fixed and permeabilized in a 96 well plate. After blocking, antibodies against phosphorylated rpS6 and total rpS6 were used in conjunction with PLA rabbit and mouse probes to allow specific readout of rpS6 activation in single cells in a high-throughput manner. At least 10.000 cells were analyzed per sample. The graph summarizes the average PLA spot counts of 3 technical replicates. Error bars are SD and *P*-values were determined in one-way ANOVA and post-hoc Bonferroni multiple comparison.

(B) Ba/F3 cells were cultured in 10 ng/mL IL-3. 17 hrs before lysis, the culture medium was changed to medium containing either 10 ng/mL IL-3 or no IL-3. PLA protocol was performed like in (A). Direct interaction between RAS and phosphor-PTPN11 was measured via PLA in high-throughput microscopy. At least 2.500 cells were analyzed per sample. The graph summarizes the average PLA spot counts of 3 technical replicates. Error bars are SD and *P*-values were determined in one-way ANOVA and post-hoc Bonferroni multiple comparison.

Supplementary-Fig.S4

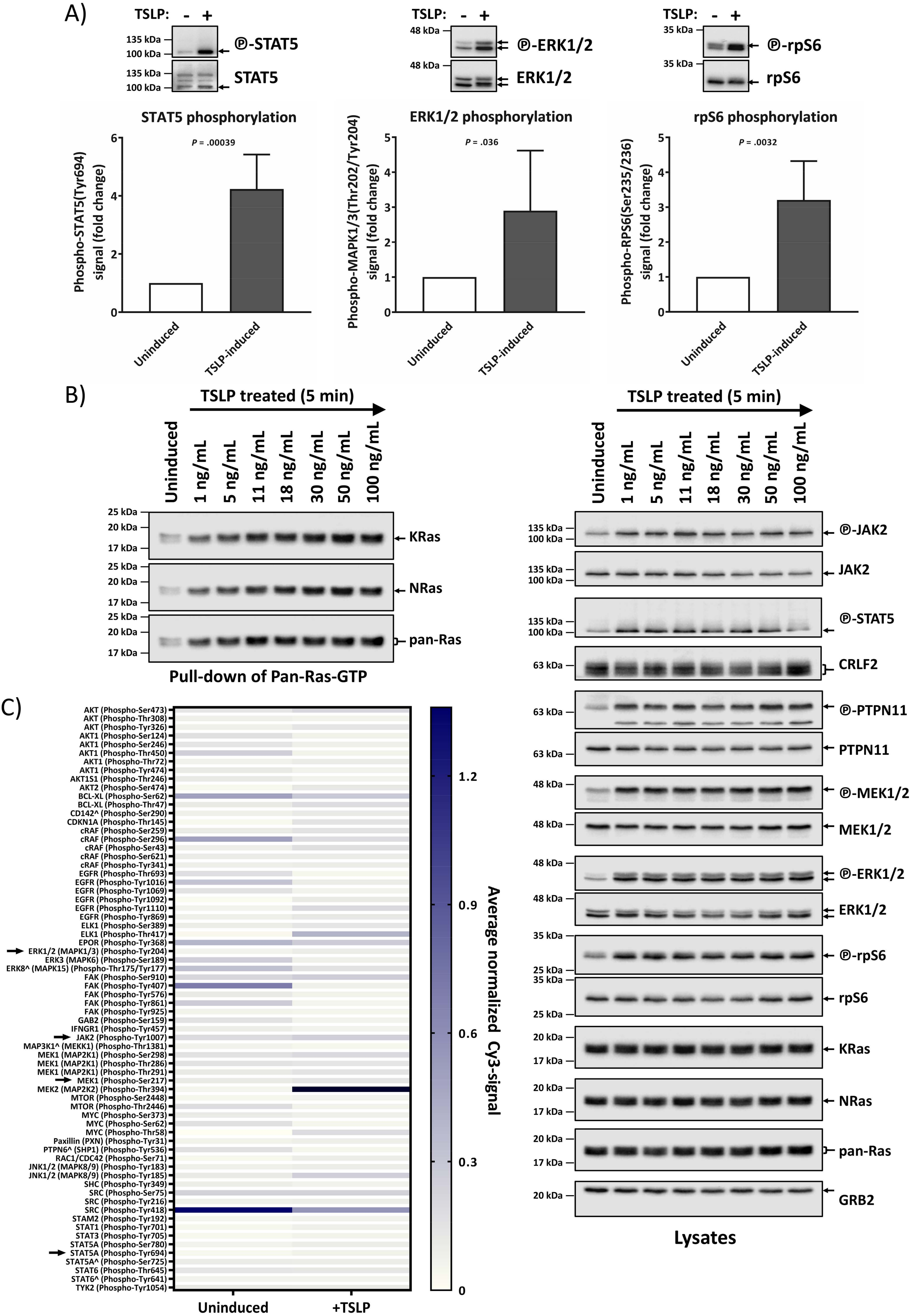

Supplementary-Fig.S4) TSLP-induction actives STAT and PI3K/mTOR signaling in Ph-like B ALL cells. Titration of TSLP concentration in Ph-like B ALL cells. (A) Extension of Fig.2A. MUTZ-5 cells (Human Ph-like B-ALL cells bearing CRLF2-rearranged and spontaneous JAK2R683G mutation) were stimulated (or not) with 20 ng/mL human TSLP for 10 min before cell lysis. Lysates were loaded on an SDS-PAGE gel followed by Western blotting. To assess the total protein and phosphorylated protein amounts on the same PVDF-membrane, membranes were stripped and reprobed with new antibodies. The experiment was repeated 5 times independently and the graphs show the quantification for active STAT5 (phosphorylated STAT5), active ERK1/2 (phosphorylated ERK1/2) and active RPS6 (phosphorylated RPS6). Representative Western blots above the respective graph show the phosphorylated protein form and the total expression of each protein.

(B) Effect of TSLP concentration titration. MUTZ-5 cells were incubated with the indicated amounts of TSLP (0 ng/mL to 100 ng/mL) for 5 min and then the cells were lysed on ice. Each cell lysate was split up for analysis in RAS-GTP pull-down assay and for total protein signal. RAS-GTP pull-down and lysate samples were loaded on separate gels. An SDS-PAGE followed by Western blotting was performed. To assess the total protein and phosphorylated protein amounts on the same PVDF-membrane, membranes were stripped and reprobed with new antibodies. Left-hand side blots show the RAS-GTP pull-down while the right-hand side blots show whole cell lysates of the same samples. Antibody-targets are labeled on the right side of each image with black arrows indicating the respective protein band.

(C) Whole, non-denatured lysate from uninduced or TSLP-induced (20 ng/mL, 10 min) MUTZ-5 cells was subjected to an antibody-microarray. Binding of Cy3-labeled, endogenous proteins was measured. 6 spots per antibody were averaged and normalized to the Cy3-control present on each microarray slide. To exclude changes that are due to epitope-obstruction from bound protein partners, the signal for phosphorylation-site-specific antibodies was normalized to the signal from each corresponding total antibody raised against the same (unphosphorylated) peptide sequence (for phosphorylation-sites marked with ^, no total peptide counterpart was contained on the microarray-slide). Changes in phosphorylation that originated completely from epitope-availability were disregarded from the analysis altogether. BSA-spots (empty) were used to set and subtract background signal. For analysis, minimum signal threshold was set to 3% signal strength of the Cy3-normalization control. Heatmap shows the signal for all normalized phosphorylation-sites on the antibody microarray that passed the mentioned criteria (68/97). Black arrows indicate phosphorylation-sites that were also investigated via Western blot in this work.

Supplementary-Fig.S5

A)

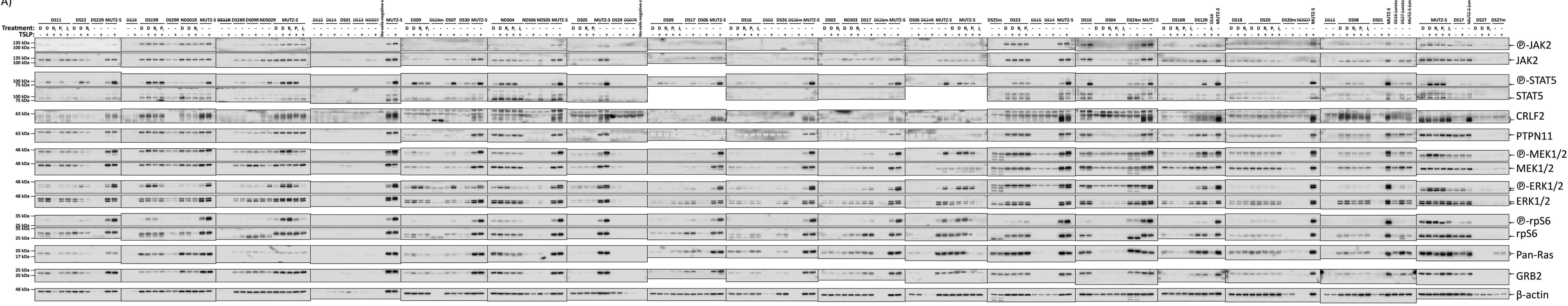

B)

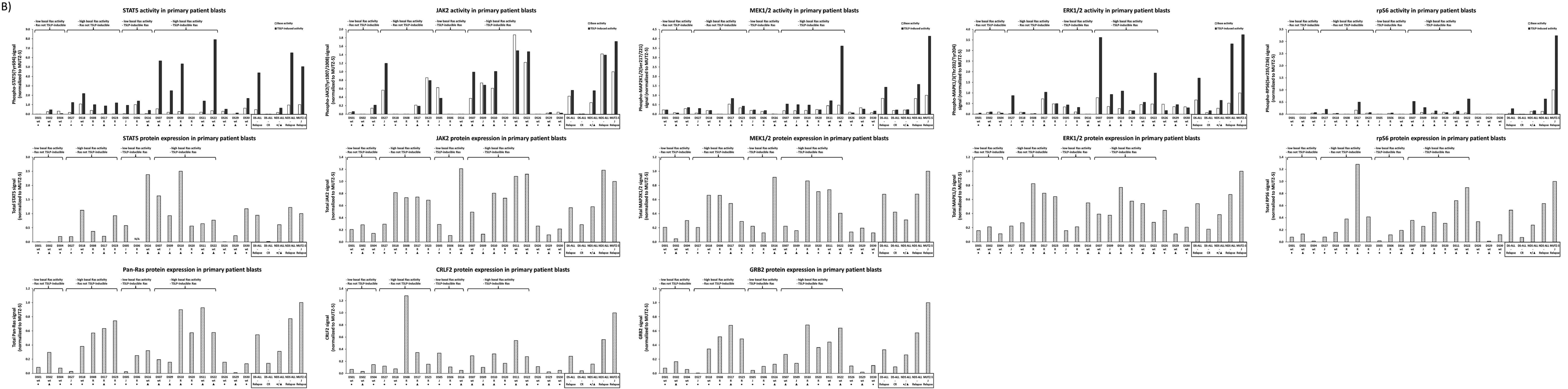

Supplementary-Fig.S5) Western blots for all analyzed patient samples and the resulting quantified data for the sub-stratification of DS-ALL by PCA. (A) Primary samples of DS-ALL patients at presentation, relapse, or remission, as well as MUTZ-5 cells, were thawed and cells were gently recovered in 9 mL RPMI1640 (Cat.#11-875-119; Life Technologies, Carlsbad, US) containing 20% characterized fetal bovine serum (FBS; Cat.#SH30071.03; GE Healthcare, Chicago, US), 20 U/mL Benzonase (Cat.#70746; EMD Millipore), 2 mM L-Glutamine (Cat.#25030081 Life Technologies), and 100 U/mL Penicillin-Streptomycin (Cat.#15140122, Life Technologies). Cells were resuspended in 5 mL fresh medium without Benzonase. Cells were counted using the automated cell-counter NucleoCounter NC-250 (ChemoMetec, Allerod, DK) and viability was assessed via staining with Solution18 (Cat.#910-3018, ChemoMetec). Cells were seeded at 1.5×10^6 cells/mL density in IMDM-complete (IMDM (Cat.#12440053; Life Technologies) with 10% FBS, 2 mM L-Glutamine, 100 U/mL Penicillin-Streptomycin, 200 µg/mL apo-Transferrin (Cat.#11096-37-0; Santa Cruz Biotechnlogy), 0.1% 2-Mercaptoethanol (Cat.#21985023; Life Technologies), 1 µg/mL insulin (Cat.#11061-68-0; Sigma-Aldrich), 10 ng/mL IL-3 (Cat.#200-03; Peprotech, Rocky Hill, US) and 10 ng/mL IL-7 (Cat.#200-07; Peprotech). After 24 hrs, surviving cells were reseeded at 1×10^6 viable cells/mL density in IMDM-complete. After 16 hrs the cells were reseeded at 1×10^6 cells/mL density in 2 mL OptiMEM (Cat.#31985070; Life Technologies) with 5% FBS. After 3 hrs, cells were either left uninduced or induced with 20 ng/mL TSLP (Cat.#1398-TS; R&D Systems) for 10 min at 37 °C before lysis. Where indicated, patient cells were pre-treated during the 3 hrs incubation with DMSO (D), RAS-inhibitor (R_i), PI3K/mTOR inhibitor (P_i), or JAK inhibitor (J_i). For Western blot analysis, cells were lysed and loaded on an SDS-PAGE gel. To assess the total protein and phosphorylated protein amounts on the same PVDF-membrane, membranes were stripped and reprobed with new antibodies. Antibody-targets are labeled on the right side of each image with black arrows indicating the respective protein band. As all lanes are shown for all gels in every specific staining, sample names that are crossed-out were either not part of the analysis, or showed too low loading in all expressed proteins to be used for any quantification. For blot imaging, each PVDF membrane piece was separately incubated with chemiluminescent HRP substrate solution (Cat.#WBKLS0500; EMD Millipore) for 2 min and imaged via the auto-exposure function of the ChemiDoc MP imaging system (Bio-Rad Laboratories) within 100 sec exposure time to standardize the sensitive but otherwise semi-quantifiable enzymatic-based detection.

(B) Quantification of Western blot signals in (A) for all DS-ALL samples at presentation as well as Non-DS (NDS) at presentation, DS complete remission (CR), and DS/NDS at relapse (boxed group at right end of each bar graph). For quantification, the raw images were analyzed in Fiji 1.52n (ImageJ, National Institutes of Health, US) using the subtract background process followed by measuring the signal peaks in the Gel Analyzer function. Microsoft Excel software was used to adjust all samples to either their loading control (β-actin) or the quantitative total protein fluorescent signal (AzureRed, Cat.#AC2124; Azure Biosystems, Dublin, US). All samples' protein activity and total protein signals were normalized to the respective signal from the uninduced MUTZ-5 samples loaded as reference on each membrane. 81% of all analyzed samples showed sufficient signal in loading control and tested proteins to be appropriate for further normalization and analysis. The quantified phosphorylated protein signals were additionally adjusted to the respective quantified total protein loading. Brackets on top indicate the groups of the four RAS activity patterns presented in Fig.3C. White and black bar graphs show the basal and TSLP-induced activation levels, respectively, of STAT5, JAK2, MEK1/2, ERK1/2, and RPS6 while the dotted grey bar graphs show the respective total protein expression levels. Outcome of leukemia is symbolized as circle for good outcome and triangle for poor outcome; RAS pathway mutations (R), JAK2 mutations (J), or neither (wt) are listed for each DS-ALL patient.

Supplementary Fig.S6

A)

Field	PC1	PC2	PC3
Dispers	0.385601	0.049523	0.081659
Overall	0.385601	0.435125	0.516784
Pan-Ras basal activity	0.214594	**0.485179**	0.048106
Pan-Ras activity with TSLP	0.227889	**0.474698**	0.041787
JAK2 basal activity	0.237394	-0.24357	-0.40745
JAK2 activity with TSLP	0.297418	-0.1813	-0.35807
STAT5 basal activity	0.172951	-0.20932	0.065401
STAT5 activity with TSLP	**0.322827**	0.163583	-0.23887
MEK basal activity	0.311386	0.309735	0.091045
MEK activity with TSLP	**0.351922**	0.1494	0.076544
ERK basal activity	0.281839	-0.06334	-0.04313
ERK activity with TSLP	0.287373	-0.22426	-0.26412
S6 basal activity	0.226173	-0.24303	**0.609553**
S6 activity with TSLP	0.280702	-0.32844	**0.303667**
CRLF2 protein expression	0.250139	-0.18761	0.007298

D)

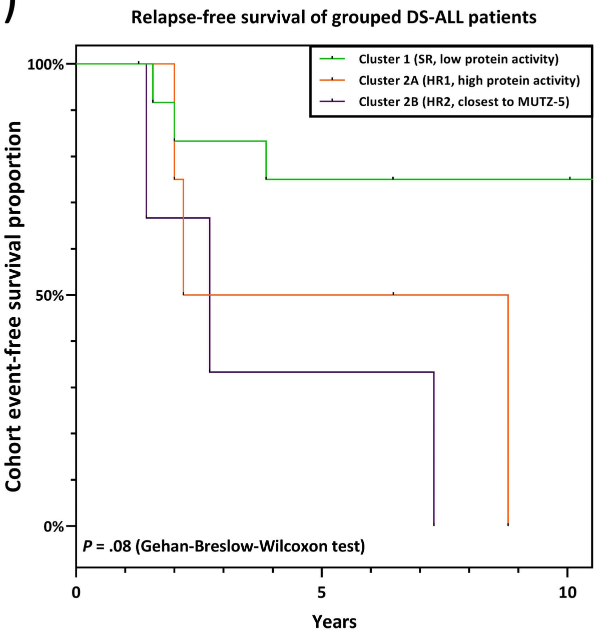

B)

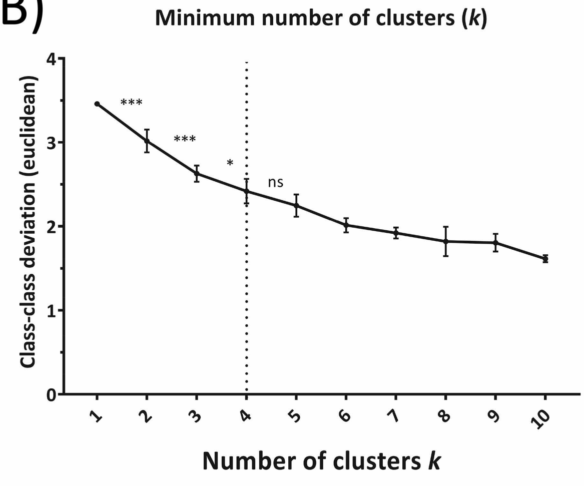

E)

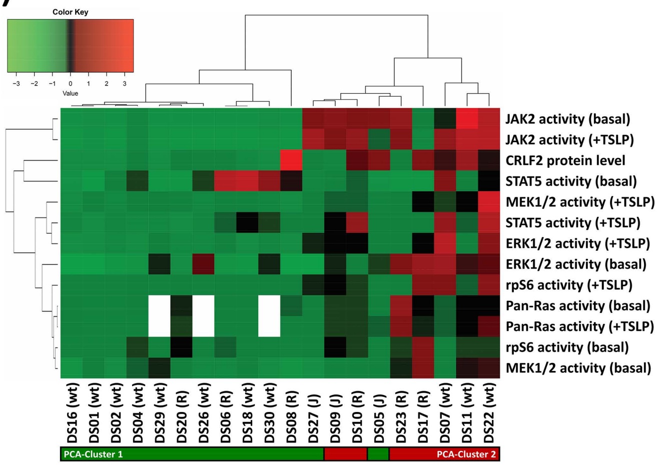

C)

	N =	DS patients with good outcome	DS patients with poor outcome	DS relapse samples	DS Complete remission sample	DS Remission sample with imminent relapse	NDS patients with good outcome	NDS patients with poor outcome	NDS relapse samples	NDS (Ph-like) relapse cell line (MUTZ-5)
**Total**	**38**	**11**	**9**	**7**	**2**	**2**	**3**	**1**	**2**	**1**
Cluster 1	19	10	3	1	2	0	2	1	0	0
Cluster 2	15	1	6	4	0	2	1	0	1	0
Cluster 3	3	0	0	1	0	0	0	0	1	1
Cluster 4	1	0	0	1	0	0	0	0	0	0

Supplementary-Fig.S6) PCA statistics and PCA cluster counts on primary DS-ALL and NDS-ALL blast cells. (A) Basal and induced activation levels for RAS, JAK2, STAT5, MEK, ERK, and RPS6, as well as CRLF2 protein expression, were fed into ViDaExpert v1.2 to calculate 3 main principal components (PC1-3) in a PCA analysis. Numbers in bold highlight the categories (field) with the greatest correlation within each PC.

(B) Euclidean *k*-means clustering was performed in ViDaExpert v1.2 on the PCA analysis in (A) for *k* = 1 to 10 (6 times each). In order to identify the minimal number of clusters needed for the grouping analysis, class-class deviation was averaged for each k (error bars are SD). Class-class deviation from sequential, increasing number of clusters stopped being statistically significant at *k* = 4 (doted line); * ($P < .05$), *** ($P < .001$), ns (not significant).

(C) Number of samples within each of the four clusters identified in the *k*-means clustering of the PCA data represented in Fig.4A. Count of the different patient sample categories for each cluster.

(D) Kaplan–Meier curves of DS-ALL patients. For this comparison, and cluster 2 (HR = high risk, see Fig.5) was split into two subclusters 2A and 2B (cluster 2B contains the DS-ALL presentation samples that grouped closest to MUTZ-5 in the PCA-based cluster analysis in Fig.5A; DS07, DS11, and DS22). Median survival for subcluster 2A was 5.49 and for 2B 2.72.

(E) Unsupervised hierarchical clustering of all analyzed DS-ALL presentation samples for all 6 measured protein activities (basal and TSLP-induced) as well as CRLF2 protein expression. The clustering results were similar to the independent *K*-means clustering (indicated by the red/green colored bar underneath the sample IDs) of the PCA (Fig.4A), with 90% (18/20) of the samples clustered in the same groups by both methods. Presence of RAS pathway mutations (R), JAK2 mutations (J), or neither (wt) is indicated (no single patient in this cohort was found to have both RAS and JAK2 mutations).

Supplementary-Fig.S7

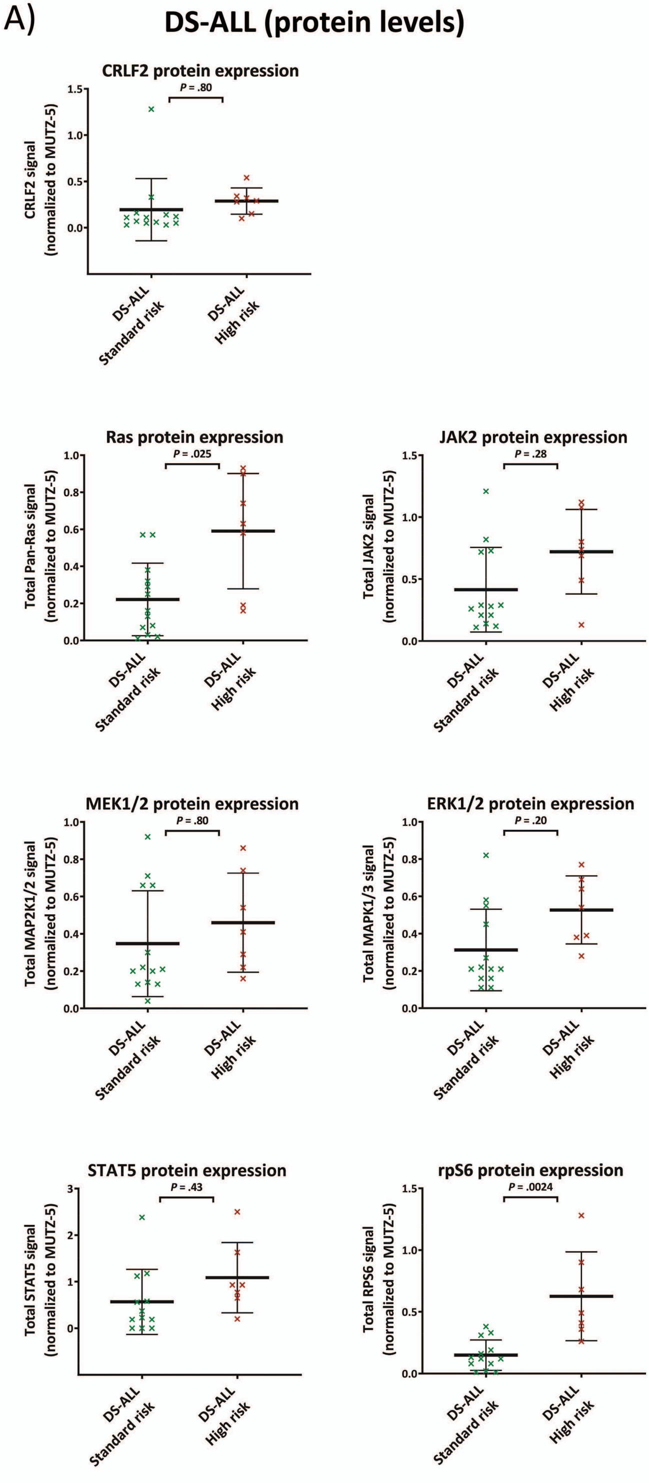

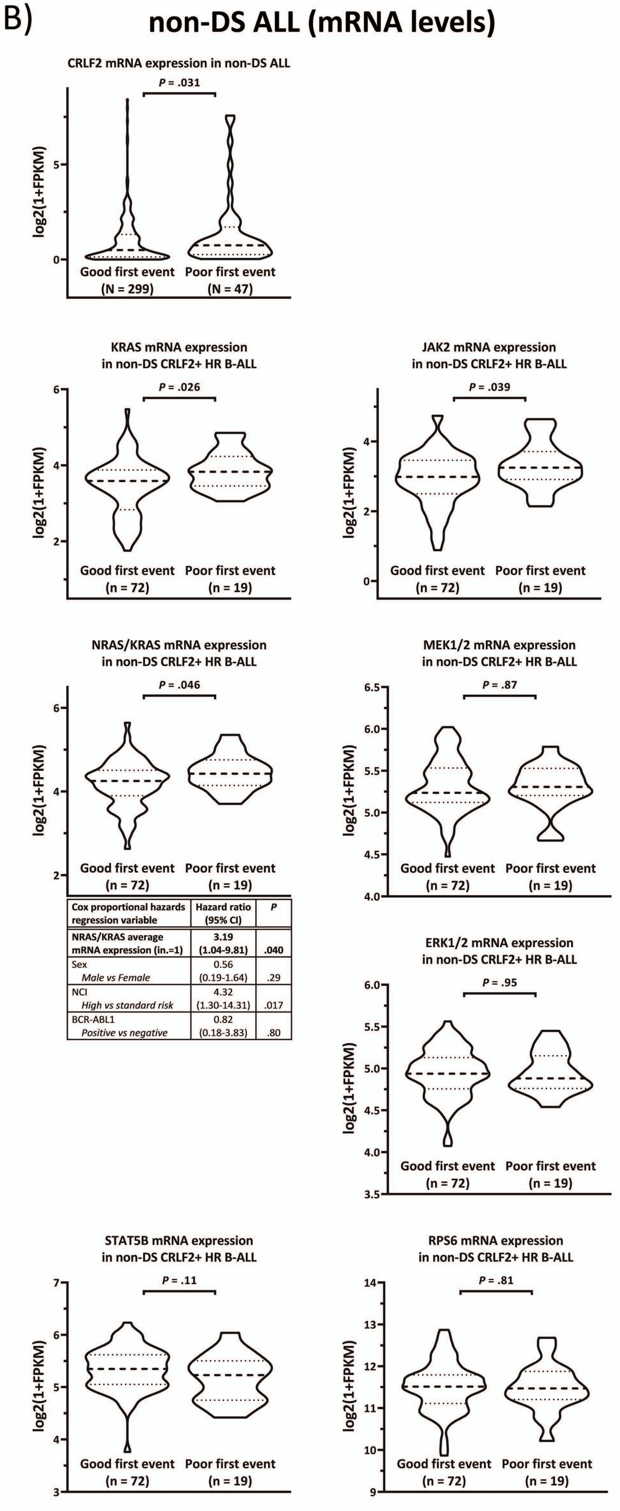

Cox proportional hazards regression variable	Hazard ratio (95% CI)	*P*
**NRAS/KRAS average mRNA expression (in.=1)**	**3.19 (1.04-9.81)**	**.040**
Sex *Male vs Female*	0.56 (0.19-1.64)	.29
NCI *High vs standard risk*	4.32 (1.30-14.31)	.017
BCR-ABL1 *Positive vs negative*	0.82 (0.18-3.83)	.80

Supplementary-Fig.S7) Disease outcome-correlations with expression levels of components used in RAS-pathway sub-stratification. Protein levels in DS-ALL (A) and mRNA levels in non-DS childhood ALL (MS2003/2010 cohorts) (B)

(A) Average protein expressions of analyzed key pathway components are compared between the SR group (DS-ALL patients at diagnosis in PCA cluster 1) and the HR group (DS-ALL patients at diagnosis in PCA cluster 2) identified in Fig.4. All error bars are SD. *P*-values shown are Student's T-test after Bonferroni correction for sequential multiple comparison for all proteins analyzed.

(B) Violin blots of the mRNA levels based on whole transcriptome RNAseq of the MS2003/2010 cohorts at diagnosis are shown for gene(s) equivalents to proteins analyzed in the DS-ALL cohort. The dashed line represents the median while the dotted lines above and below the median mark the respective quartiles. *CRLF2* was analyzed for outcome on the total non-DS ALL cohort (N = 346). Subsequently, all other shown mRNA expression levels (*KRAS*, *KRAS*&*NRAS* combined, *STAT5B*, *JAK2*, *MEK1*&*2* combined, *ERK1*&*2* combined, *RPS6*) were compared in B-ALL samples positive for *CRLF2*-mRNA expression (log2(1+FPKM) > 0.7) for the first event outcome. Poor first event for the MS2003/2010 cohort was defined as resistance, death or relapse; good first event means complete remission. Subtypes that are known to favor a good outcome were excluded from the analysis (ETV6-RUNX1, hyperdiploid, DUX4 and ZNF384). The resulting n=91 subcohort harbored 13 *RAS* mutations (4/19 poor outcome samples) and 6 *JAK*2 mutations (0/19 poor outcome patients) but never both together. For *KRAS*&*NRAS* combined, table details results of Cox proportional hazards regression. All error bars are SD. *P*-values shown were calculated using Student's T-test. Bonferroni-*P*-values adjusted for sequential multiple comparison were not ≤ α (0.05) and are listed in Supplementary-Tab.S2.

Supplementary-Fig.S8

A) Non-DS ALL mRNA levels (full MS2003/2010 cohort)

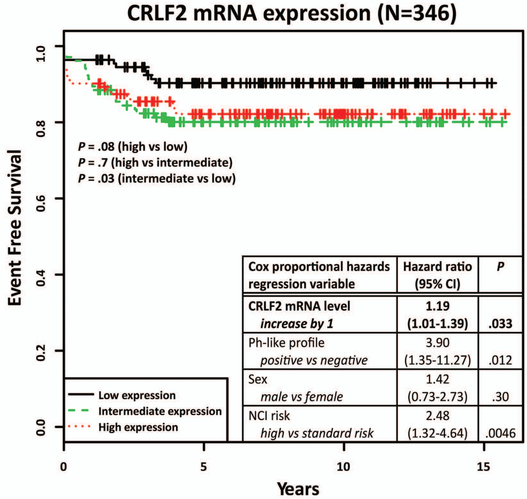

Cox proportional hazards regression variable	Hazard ratio (95% CI)	*P*
**CRLF2 mRNA level** ***increase by 1***	**1.19 (1.01-1.39)**	**.033**
Ph-like profile *positive vs negative*	3.90 (1.35-11.27)	.012
Sex *male vs female*	1.42 (0.73-2.73)	.30
NCI risk *high vs standard risk*	2.48 (1.32-4.64)	.0046

Number at risk:																
Low expression	111	107	98	89	83	71	65	58	47	40	33	24	14	6	4	2
Interm. expression	104	94	84	78	63	55	51	44	37	30	26	16	15	10	5	3
High expression	112	101	92	84	76	70	66	57	47	44	31	23	18	12	4	2

B) Non-DS ALL mRNA levels (CRLF2+, high-risk genetics subcohort)

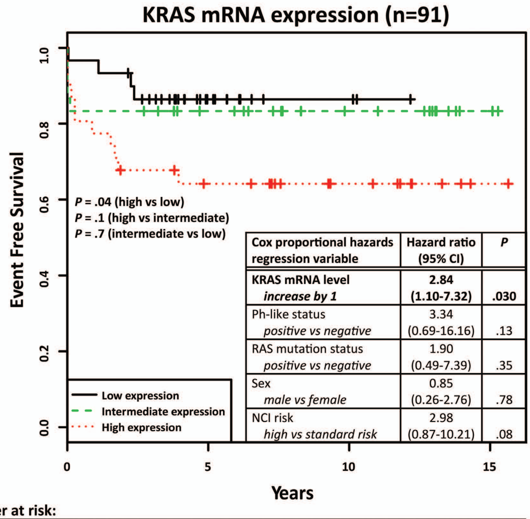

Cox proportional hazards regression variable	Hazard ratio (95% CI)	*P*
**KRAS mRNA level** ***increase by 1***	**2.84 (1.10-7.32)**	**.030**
Ph-like status *positive vs negative*	3.34 (0.69-16.16)	.13
RAS mutation status *positive vs negative*	1.90 (0.49-7.39)	.35
Sex *male vs female*	0.85 (0.26-2.76)	.78
NCI risk *high vs standard risk*	2.98 (0.87-10.21)	.08

Number at risk:																
Low expression	30	29	28	23	17	11	7	3	3	3	3	1	1	0	0	0
Interm. expression	30	25	25	24	20	19	18	16	13	12	11	11	10	7	2	2
High expression	31	24	20	20	18	17	17	16	12	12	9	8	6	4	2	1

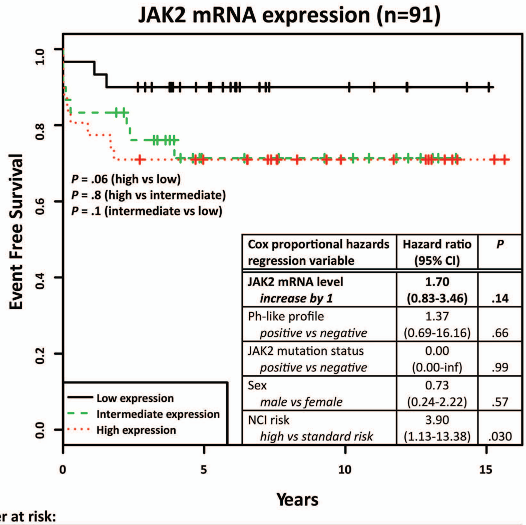

Cox proportional hazards regression variable	Hazard ratio (95% CI)	*P*
**JAK2 mRNA level** ***increase by 1***	**1.70 (0.83-3.46)**	**.14**
Ph-like profile *positive vs negative*	1.37 (0.69-16.16)	.66
JAK2 mutation status *positive vs negative*	0.00 (0.00-inf)	.99
Sex *male vs female*	0.73 (0.24-2.22)	.57
NCI risk *high vs standard risk*	3.90 (1.13-13.38)	.030

Number at risk:																
Low expression	30	29	27	25	19	17	12	8	6	6	6	5	4	2	2	1
Interm. expression	30	25	24	21	15	11	11	10	9	9	8	6	5	3	0	0
High expression	31	24	22	21	21	19	19	17	13	12	9	9	8	6	2	2

Supplementary-Fig.S8) Disease outcome-correlations and multivariate analysis for mRNA expression levels in non-DS childhood ALL (MS2003/2010 cohorts) of key components emanating from RAS-pathway sub-stratification in primary samples at diagnosis (A) Kaplan–Meier estimates of event-free survival of all non-DS ALL patients (N=346) according to *CRLF2*-mRNA expression. Low, intermediate, and high curves for *CRLF2*-mRNA expression levels (overall P = .09) each represent one third of the cohort (bottom, mid, and top third, respectively). Table within the graph shows multivariate analysis using Cox proportional-hazards model for *CRLF2*-mRNA levels together with the prognostic factors Ph-like status, sex, and NCI risk groups. Patient numbers at risk for each year are given in the table below the survival curve.

(B) Subsequently, all other mRNA expression levels (*KRAS*, JAK2, *KRAS*&*NRAS* combined, *STAT5B*, *MEK1*&*2* combined, *ERK1*&*2* combined, *RPS6*) were compared in B-ALL samples positive for *CRLF2*-mRNA expression (log2(1+FPKM) > 0.7) for the first event outcome (Supplementary-Fig.S7B). Subtypes that are known to favor a good outcome were excluded from the analysis (ETV6-RUNX1, hyperdiploid, TCF3-PBX1, DUX4, and ZNF384). The resulting n=91 subcohort harbored 13 *RAS* mutations (4/19 poor outcome samples) and 6 *JAK2* mutations (0/19 poor outcome patients), but never both together. Kaplan–Meier curves show estimates of event-free survival of the HR non-DS ALL subcohort (n=91) grouped by *RAS* (overall P = .08) or *JAK2*-mRNA (overall P = .2) expression levels. The tables within the graphs show multivariate analysis using Cox proportional-hazards model for either *RAS*-mRNA or *JAK2*-mRNA levels respectively, together with *KRAS/NRAS* or *JAK2* activating mutations respectively, and the prognostic factors Ph-like status, sex, and NCI risk groups.

Supplementary-Fig.S9

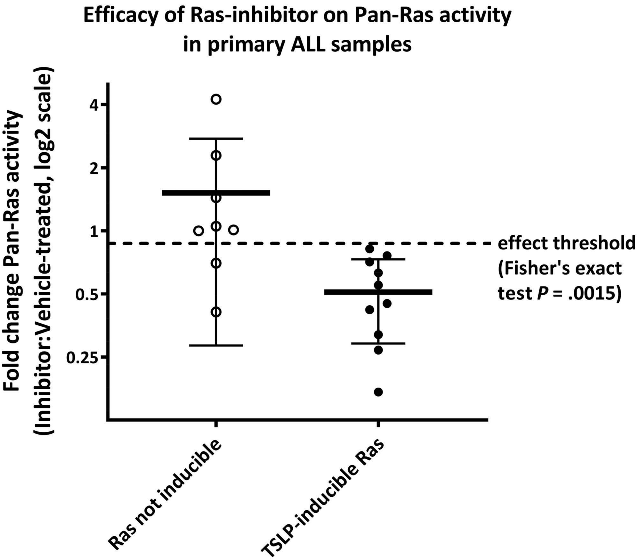

Supplementary-Fig.S9) Efficacy of the RAS inhibitor stratified by patient sample inducibility. Primary presentation samples of DS-ALL and non-DS ALL patients (characterized in Supplementary-Tab.S1) were cultured for 2 days (see Supplementary-Fig.S5A for details). Samples with sufficient cell count were treated with either 0.5% DMSO (vehicle control) or 50 µM Salirasib (indirect pan-RAS inh.) for 3 hrs after which the cells were induced for 10 min with 20 ng/mL TSLP in serum-reduced medium. Cells were lysed and a RAS-GTP ELISA pull-down assay was performed. The efficacy of the RAS inhibitor (expressed as RAS activity of inhibitor-treated divided by RAS activity of DMSO-treated) on ELISA-measured RAS activity in patient samples that were defined in Fig.3B as not TSLP-inducible for RAS is compared to those in which RAS activity was inducible by TSLP. If inhibitor treatment reduced the RAS activity by over 10% compared to vehicle-control (dashed line in plot), the sample was tallied as successful RAS blocking. A Fisher's exact test was performed between the groups.
